# Integrated Optimization of *Opuntia stricta* Extracts Coupled with HPLC-Based Phenolic Profiling: Linking Phytochemical Composition to Molecular Docking Simulation

**DOI:** 10.3390/foods15193557

**Published:** 2026-10-07

**Authors:** Moufida Chaari, Sarra Akermi, Khaoula Elhadef, Walid Elfalleh, Hassan Ahmed Rudayni, Slim Smaoui

**Affiliations:** 1 Laboratory of Microbial Biotechnology and Engineering Enzymes (LMBEE), Center of Biotechnology of Sfax (CBS), University of Sfax, Road of Sidi Mansour Km 6, P.O. Box 1177, Sfax 3018, Tunisia; moufida.chaari97@gmail.com (M.C.); sarahakermi221@gmail.com (S.A.);; 2 Department of Biology, College of Science, Imam Mohammad Ibn Saud Islamic University (IMSIU), Riyadh 11623, Saudi Arabia; harudayni@imamu.edu.sa

**Keywords:** *Opuntia stricta*, Box–Behnken Design, optimization, phytochemical contents, antioxidant activity, antibacterial activity, molecular docking, polyphosphate kinase, fibronectin binding protein A

## Abstract

This study intended to simultaneously model and optimize aqueous extracts obtained from whole *Opuntia stricta* fruits (OSE) to maximize the overall response profile, namely total phenolic content (TPC), total flavonoid content (TFC), antioxidant activity and antibacterial efficacy. For this purpose, the Response Surface Methodology was used to evaluate the influence of three extraction parameters: temperature, sample-to-solvent ratio, and time. Optimal extraction was predicted at 40 °C, 35.6 min, and a 1:50 (g/mL) ratio, yielding predicted TPC, TFC, ABTS IC_50_, and MIC values of 77.194 mg GAE/g, 3.787 mg CE/g, 0.187 mg/mL, 5.476 mg/mL, and 7.883 mg/mL, respectively. Experimental validation gave 77.23 mg GAE/g, 3.74 mg CE/g, 0.19 mg/mL, and MICs of 5 and 10 mg/mL against *S. aureus* and *E. coli*, respectively. Good agreement was observed between predicted and experimental values for most responses (relative errors: 0.05–9.52%), whereas anti-*E. coli* activity showed lower predictive accuracy, with a relative error of 21.17%. Principal component analysis (PCA) differentiated the various OSEs and revealed associations between their phytochemical composition and biological activities. High-performance liquid chromatography analysis of the optimized OSE revealed that 4-hydroxyphenylacetic acid was the predominant phenolic acid identified, while epicatechin was the major flavonoid detected. Molecular docking calculations revealed that catechin exhibited the most negative free energy of binding towards the studied virulence factors, including PPK (−8.2 Kcal/mol) and Fibronectin-binding protein A (−7.9 Kcal/mol) of *E. coli* and *S. aureus*, respectively. These findings indicate notable target interactions and highlight the bioactive potential of OSE, supporting further in vitro/in vivo validation and functional and industrial applications.

## 1. Introduction

The growing consumer demand for “clean label” products and natural food additives has significantly intensified research into plant-derived bioactive compounds as alternatives to synthetic preservatives [1,2]. In this vein, fruits rich in phenolic and flavonoid compounds have attracted increasing attention for their multifunctional properties, particularly their antioxidant and antimicrobial activities, which can enhance food stability, safety, and nutritional quality [3,4,5,6]. Among these promising plant resources, species of the genus *Opuntia* have emerged as valuable sources of functional phytochemicals, attracting increasing interest for both food and health-related applications [7,8,9]. *Opuntia stricta*, belonging to the *Cactaceae* family, is widely distributed in arid and semi-arid regions and produces fruits that remain largely neglected and underexploited despite their nutritional richness and functional value [10,11]. Studies focusing on the characterization of *Opuntia stricta* fruit remain underexplored compared with those on more widely cultivated species such as *Opuntia ficus*-indica [12,13]. This is mainly because the plant is considered wild or invasive in many areas, which limits its large-scale agricultural production [14]. Its use remains mostly limited to local consumption, which reduces its commercial importance [14]. The fruits are generally collected and consumed informally rather than processed on an industrial scale. Due to its diverse phytochemical compounds, many investigations have reported the strong antioxidant and antibacterial potential of *Opuntia* extracts from different parts, including fruits, seeds, and cladodes [10,14,15]. Among these compounds are betalains, which are well known for their remarkable free radical scavenging capacity [16,17]. *Opuntia* fruits also contain a wide range of phenolic acids and flavonoids, such as gallic acid, vanillic acid, quinic acid, catechin, rutin, hyperoside, and quercetin 3-O-rutinoside, which contribute to their high antioxidant and antibacterial activities [18,19,20,21]. The diversity and abundance of these phytochemicals highlight their potential use as a valuable natural source of health-promoting compounds [22,23]. However, this composition can change depending on several factors. One important factor is the part of the plant used, such as fruits, peels, or cladodes [20,24]. Another key factor is the extraction method and parameters [25,26]. While several investigations have studied the extraction and characterization of betalains from *Opuntia stricta*, partial attention has been dedicated to the simultaneous optimization of phytochemical composition and biological activities. However, previous works on *Opuntia* species have shown the effectiveness of Response Surface Methodology (RSM) using Box–Behnken designs for the optimization of extraction conditions of bioactive compounds, such as the studies of Vázquez-Espinosa et al. [27], who optimized the ultrasound-assisted extraction of betalain-rich compounds from *Opuntia dillenii*. They studied several key parameters, including ethanol percentage, extraction temperature, ultrasound amplitude, and cycle [27]. The optimization process was carried out by considering total betalain recovery and total antioxidant capacity as the main responses. In another study, a Box–Behnken design within Response Surface Methodology was applied to optimize the extraction of betalain-rich bioactive compounds from the fruit of *Opuntia ficus*-indica [28]. The optimization was based on total betalain content, total phenolic content, and total antioxidant capacity [28]. Building on this approach, the present study was undertaken to design an optimized extraction approach targeted at simultaneously exploiting phytochemical content and bioactive performance, thereby enhancing the overall functional value of *Opuntia stricta* extracts. In this context, RSM based on a Box–Behnken experimental design was applied to optimize the extraction conditions of bioactive compounds from the whole fruit extract of *Opuntia stricta* (OS). Additionally, a molecular docking approach was carried out to highlight its antibacterial potential and to predict possible interactions with selected bacterial pathways.

## 2. Materials and Methods

### 2.1. Reagents

Folin–Ciocalteu reagent (0.2 N) and aluminum chloride (AlCl_3_) were purchased from Loba Chemie (Mumbai, India). Sodium carbonate (Na_2_CO_3_), ABTS reagent, and MTT solution [3-(4,5-dimethylthiazol-2-yl)-2,5-diphenyltetrazolium bromide] were obtained from Bio-Basic Inc. (Markham, ON, Canada). Sodium hydroxide (NaOH) was supplied by Central Drug House (CDH, New Delhi, India). Ascorbic acid (>99%) was purchased from Sigma-Aldrich (St. Louis, MO, USA), while gallic acid (98%) was obtained from Fluka.

All phenolic compounds used as standards: chlorogenic acid, coumaric acid, 4-hydroxyphenylacetic acid (HPAC), caffeic acid, vanillic acid, 3,4-dihydroxyphenylacetic acid (DOHPAC), trans cinnamic acid, catechin, catechol, epicatechin, rutin, apigenin, protocatechic acid, tyrosol, esculin, and esculetin were purchased from Sigma–Aldrich with high purity (≥95%).

### 2.2. Plant Material

The prickly pear fruits (*Opuntia stricta*) used in this study were collected in February 2026 from the Chaffar forest in the city of Sfax, Tunisia (34°32′50.2” N 10°35′26.9” E). The fruits were washed with distilled water, then cut and ground. They were subsequently dried at 40 °C in an oven until a constant weight was achieved. After drying, the samples were ground again using a grinder, then sieved and stored in light-protected containers until use.

### 2.3. Optimization of Extraction Using Response Surface Methodology (RSM): Box–Behnken Design

The purple prickly pear (*Opuntia stricta*) extract was prepared by maceration in distilled water under continuous agitation at 200 rpm. The extraction parameters, including the plant material/water ratio, extraction time, and extraction temperature, were optimized using Response Surface Methodology (RSM) by applying the Box–Behnken design.

The Box–Behnken design was employed to optimize the extraction variables. The effects of three independent variables, namely temperature (X_1_), time (X_2_), and sample/solvent ratio (X_3_), were investigated at three coded levels: (−1), (0), and (+1). The sample/solvent ratios (1:50, 1:30, and 1:20, g/mL) were maintained at different temperatures (25, 32.5, and 40 °C) for varying extraction times (15, 30, and 45 min). The obtained extracts were then filtered and centrifuged at 10,000× *g* for 10 min at 4 °C. Finally, the different extracts were dried using a rotary evaporator at 40 °C. The extraction percent yield (%) was calculated as follows Equation (1):

(1) $$\mathrm{Yield}\left(\%\right)=\frac{\mathrm{the}\mathrm{mass}\mathrm{ot}\mathrm{the}\mathrm{extract}\mathrm{after}\mathrm{solvent}\mathrm{evaporation}}{\mathrm{the}\mathrm{mass}\mathrm{of}\mathrm{the}\mathrm{dry}\mathrm{plant}\mathrm{material}}\times 100$$

A total of 15 experimental runs (Table 1) were carried out to optimize total phenolic compounds, total flavonoids, antioxidant activity (ABTS assay), and antibacterial activity against *Staphylococcus aureus* and *Escherichia coli*.

Two-dimensional (2D) contour plots were generated to illustrate the relationships between the measured responses and the experimental levels of the independent variables. Process optimization was performed using the Response Optimizer tool in Minitab^®^ version 22. The responses corresponding to total phenolic content (TPC) and total flavonoid content (TFC) were set to be maximized, whereas antioxidant and antibacterial activity values were set to be minimized, since lower IC_50_ and MIC values indicate stronger antioxidant and antibacterial activities, respectively. The overall desirability function was calculated by combining the individual desirability values of each response to simultaneously identify the optimal extraction conditions.

### 2.4. Determination of Total Phenolic Content (TPC)

The total phenolic content of the OS extracts was quantified following the procedure reported by Zugazua-Ganado et al. [29]. Briefly, 100 µL of OS extract solution at 10 mg/mL was combined with 400 µL of Folin–Ciocalteu reagent (0.2 N). Subsequently, 500 µL of sodium carbonate solution (7.5% Na_2_CO_3_) was added to the mixture. After incubation for 2 h at room temperature in the absence of light, the absorbance was recorded at 765 nm. Gallic acid was employed as the calibration standard within the range of 0–0.2 mg/mL, and its absorbance was measured at 765 nm. The results were expressed as milligrams of gallic acid equivalents per gram of extract (mg GAE/g).

### 2.5. Determination of Total Flavonoid Content (TFC)

The total flavonoid content was evaluated using the aluminum chloride (AlCl_3_) colorimetric assay according to the method described by Ghasemzadeh et al. [30]. Initially, 500 µL of OS extract solution at 10 mg/mL was mixed with 2 mL of distilled water, followed by the addition of 150 µL of sodium nitrite solution (15%). After 6 min, 150 µL of 10% AlCl_3_ solution was incorporated, and the mixture was incubated again for an additional 6 min. Thereafter, 2 mL of sodium hydroxide solution (4% NaOH), together with 200 µL of distilled water, was added. The reaction mixture was vortexed thoroughly and left to stand for 15 min before measuring the absorbance at 510 nm. Catechin served as the reference compound for the calibration curve, and the flavonoid content was expressed as milligrams of catechin equivalents per gram of extract (mg CE/g extract).

### 2.6. Evaluation of Antioxidant Activity

The antioxidant potential of the purple prickly pear extract was assessed using the ABTS radical scavenging assay as described by Re et al. [31]. For the assay, 200 µL of OS extract solution at different concentrations (10, 5, 2.5, 1.25, 0.625, 0.3125, 0.156 mg/mL) was added to 800 µL of ABTS^+^ solution (7 mM). The mixtures were incubated for 10 min at room temperature in the dark, after which the absorbance was measured at 734 nm. The control consisted of the ABTS^+^ solution mixed only with the extraction solvent without the addition of the extract. Ascorbic acid was used as a positive control. The percentage of ABTS scavenging was calculated as follows (Equation (2)):

(2) $$\mathrm{ABTS}\%=\frac{A\;\mathrm{control}-A\;\mathrm{sample}}{A\;\mathrm{control}}\times 100$$

where *A control* and *A sample* correspond to the absorbance values measured for the control and the sample, respectively.

The data are expressed as the inhibitory activity of the different extracts required to reduce the ABTS absorbance to 50% inhibitory concentration IC_50_.

### 2.7. Evaluation of Antibacterial Activity by Minimum Inhibitory Concentration (MIC)

The antibacterial activity of the purple prickly pear extract was investigated by determining the minimum inhibitory concentration (MIC) according to the method reported by Fourati et al. [32]. The MIC is defined as the lowest concentration of extract capable of completely suppressing visible bacterial growth. The experiments were performed against two reference bacterial strains: *Staphylococcus aureus* ATCC 25923, a Gram-positive bacterium, and *Escherichia coli* ATCC 25922, a Gram-negative bacterium. Before testing, bacterial strains were cultured in nutrient broth and incubated at 37 °C for 18–24 h to obtain fresh cultures. The bacterial suspensions were then adjusted to approximately 10^5^ CFU/mL.

The antibacterial assay was conducted using sterile 96-well microplates. Initially, each well received 100 μL of sterile Luria–Bertani (LB) medium. Afterwards, 100 μL of OS extract at an initial concentration of 50 mg/mL was introduced into the first well of each row. Serial two-fold dilutions were subsequently prepared to obtain a range of extract concentrations. Following dilution preparation, 10 μL of bacterial suspension was added to each well. A growth control containing only culture medium and bacterial suspension was included to confirm normal bacterial growth. Finally, the microplates were incubated under sterile conditions at 37 °C for 24 h. To evaluate bacterial growth, 15 μL of MTT solution (3-(4,5-dimethylthiazol-2-yl)-2,5-diphenyltetrazolium bromide) at a concentration of 0.5 mg/mL was added to each well, followed by incubation for 15 min. This indicator produces a blue coloration in wells where bacterial growth occurs, whereas a yellow coloration indicates inhibition of microbial growth.

For the experimental validation of the predicted optimum, the antibacterial assay was performed separately using an initial extract concentration of 20 mg/mL, followed by the same two-fold serial dilution procedure.

### 2.8. High-Performance Liquid Chromatography Coupled with a Diode Array Detector (HPLC-DAD)

The analysis of phenolic compounds was carried out using an HPLC–DAD (Agilent 1260 infinity, Agilent Technologies, Waldbronn, Germany). A volume of 10 µL of OS extract was injected at a flow rate of 0.5 mL/min. Separation was performed on a C18 column (Zorbax Eclipse XDB-C18, 4.6 × 250 mm, 5 µm) maintained at 40 °C. Detection was carried out at wavelengths of 254 nm, 280 nm, and 330 nm. The mobile phase consisted of eluent A (pure water) and eluent B (100% HPLC-grade acetonitrile), using a gradient program from 10% to 100% B over 32 min, followed by a hold at 100% B until 40 min, and then a return to 10% B for re-equilibration. Quantification was conducted using an external standard curve of chlorogenic acid, coumaric acid, 4-hydroxyphenylacetic acid (HPAC), vanillic acid, 3,4-dihydroxyphenylacetic acid (DOHPAC), trans cinnamic acid, catechin, catechol, epicatechin, rutin, apigenin, protocatechic acid, caffeic acid, tyrosol, esculin, and esculetin based on their respective commercial standards (Figures S1–S15).

### 2.9. Computational Prediction of the Antibacterial Potential of OS Extract Compounds

All compounds were subjected to molecular docking calculations to predict possible interactions of OS extract with vital bacterial targets. Two bacterial virulence factors were selected: polyphosphate kinase (PPK) of *E. coli* and fibronectin-binding protein A (FnBPA) of *S. aureus.*

#### 2.9.1. Receptor Preparation

The target PPK (PDB ID: 1XDO) was obtained from the NCBI database (https://ncbi.nlm.nih.gov/?hl=ko) (Accessed on 28 July 2026). It is an experimental structure obtained by the X-ray diffraction method with a resolution of 3 Å. On the other hand, fibronectin-binding protein A (UniProt accession: P14738) was retrieved from the UniProt database (https://www.uniprot.org/) (Accessed on 28 July 2026). The latter corresponds to a short FnBPA peptide from the fibronectin-binding protein A of *S. aureus* (strain NCTC 8325/PS 47). It is represented by an experimental fragment, as no full-length experimental structure is currently available. Additionally, all crystallographic water molecules and non-essential heteroatoms were removed from the receptor structures and the FnBPA FASTA sequence was submitted to the Swiss-Model server (https://swissmodel.expasy.org/) (Accessed on 29 July 2026) for automatic homology modeling using the default parameters. The obtained protein model was subjected to model validation using the ProFunc Server (http://www.ebi.ac.uk/thornton-srv/databases/ProFunc) (Accessed on 29 July 2026) to analyse the Ramachandran plot.

#### 2.9.2. Ligand Preparation

All the compounds of the OS extract were obtained from the PubChem database (https://pubchem.ncbi.nlm.nih.gov/) (Accessed on 30 July 2026). Their SMILES strings were converted into 3D structures using the Corina Demo server (https://demos.mn-am.com/corina.html) (Accessed on 30 July 2026). All files were saved in PDB format. Subsequently, the generated structures were optimised using the software AVOGADRO version 2.0.0.

#### 2.9.3. Binding Site Prediction

The receptor structures were analysed for potential binding regions using the ProFunc server. The query structures were compared with proteins of known ligand-binding residues through the “reverse template comparison vs. PDB structures” strategy implemented in the SiteSeer module. Binding-site residues were identified from the top-scoring matches displaying an E-value of 0 and reflecting strong similarity between the studied proteins and the templates.

#### 2.9.4. Molecular Docking Simulation

The simulations were performed using AutoDock Vina version 1.2.0, following the methodology previously described by Akermi et al. [33]. In this sense, all ligand PDBQT files were generated using AutoDockTools version 1.5.7. Hydrogen atoms were added, nonpolar hydrogens were merged, and rotatable bonds were assigned according to the torsional degrees of freedom of each compound. Receptor preparation was also carried out via AutoDockTools 1.5.7 by adding all hydrogen atoms, merging nonpolar hydrogens, and assigning Gasteiger and Kollman charges. The resulting structures were saved in PDBQT format. The grid box was built around the binding site using a standard size of 60 × 60 × 60 with a spacing of 0.375 Å. The grid center coordinates were set based on the residues of the binding site within the proteins (X = −38.653, Y = −87.261, Z = 42.48 for PPK of *E. coli*) and (X = −1.763, Y = 3.848, Z = 8.541 for FnBPA of *S. aureus*. The conFigure file (conf) of AutoDock Vina was set with the name of the protein.pdbqt, information about center_x, center_y, center_z and box size of xyz. The exhaustiveness value was set to 100, with the number of modes (num_modes) set to 200 and energy_range to 4. All AutoDock Vina executable files were placed in the same directory as the compound and protein input files. A custom docking script was developed to automate the molecular docking workflow in AutoDock Vina. All ligand PDBQT files were listed in a ligand.txt file, which served as input for the Perl docking script. The script was configured to sequentially dock each ligand into the predicted binding site of the target protein, and all resulting output files were stored in a dedicated docking directory. For each ligand, the best docked conformation was selected based on clustering RMSD and the lowest binding energy.

#### 2.9.5. Molecular Docking Validation Protocol

To ensure the accuracy and reproducibility of the molecular docking simulations, a dual validation approach (redocking) was performed. The native co-crystallized ligand or substrate (ATP analogue present in the PPK structure of *E. coli*) was extracted from the binding pocket, redocked using the exact parameters that were previously described, and the Heavy-Atom Root-Mean-Square Deviation (RMSD) values between the predicted pose and the native crystallographic pose were calculated. The obtained RMSD values were below 2Å, which confirms the reliability of the settings. On the other hand, the FnBPA structure was generated via homology modeling from a peptide region. It also lacks a co-crystallized ligand or a target-specific inhibitor; redocking, in this case, was not structurally feasible.

#### 2.9.6. Complex Visualization

Finally, the resulting receptor-ligand complexes were analyzed, ranked according to their free energies of binding, and visualized using BIOVIA^®^ Discovery Studio^®^ version 2017 R2 (Dassault Systems BIOVIA).

### 2.10. Statistical Analysis

All measurements were achieved in triplicate. A one-way analysis of variance (ANOVA) and Tukey’s tests were carried out to determine significant differences between the responses using the Statistical Package for the Social Sciences (SPSS) software (SPSS Ltd., Woking, United Kingdom, version 27). In addition, ANOVA was performed to determine the linear, quadratic, and interaction regression coefficients (β) using Minitab statistical software version 22. As shown below, the mean values of the variables were fitted to a second-order polynomial model Equation (3):

(3) $$Y=\beta_{0}+\sum_{i=1}^{n}\beta_{i}\mathrm{Xi}+\sum_{i=1}^{n}\beta_{\mathrm{ii}}Xi^{2}+\sum_{i=1}^{n-1}\sum_{j=i+1}^{n}\beta_{\mathrm{ij}}\mathrm{XiXj}+\;\varepsilon$$

where *Y* is the response, while *β0*, *βi*, *βii* and *βij* correspond to the regression coefficients for the intercept, linear, interaction and quadratic, respectively. *Xi* and *Xj* are the independent variables, and *ε* is a random error.

Means and standard deviations were calculated. A *p*-value < 0.05 was used to assess the statistical significance of all experimental data.

Principal component analysis (PCA) was conducted to identify the possible links between phytochemical contents (TPC and TFC) of different extracts and their corresponding biological activities (antioxidant and antibacterial activities). The Ward technique and the squared Euclidean distance matrix were used to define each cluster, resulting in a dendrogram. These approaches were achieved using XLSTAT software for Windows (version 22). The PCA type was Pearson (*n*), the plot type was correlation biplot, and the coefficient was automatic.

## 3. Results and Discussion

The experimental matrix and the corresponding response data are summarized in Table 2. A total of 15 experimental runs were conducted to evaluate the combined effects of these variables within a multivariate framework.

Although extraction yield was not included among the optimized response variables, it was evaluated to assess the influence of the extraction conditions on the recovery of soluble constituents. The extraction yield of OS extracts varied significantly (*p* < 0.05) among the experimental conditions, ranging from 60.40% to 98.00%, indicating that extraction temperature, extraction time, and sample/solvent ratio markedly affected the extraction efficiency. However, the optimization strategy was focused on bioactivity-related responses rather than extraction yield, since the latter does not necessarily reflect the biological quality of the extracts.

The relationships between the independent variables (X_1_, X_2_, and X_3_) and the response parameters: total phenolic content, total flavonoid content, antioxidant capacity (ABTS assay radical scavenging activity), as well as antibacterial activity against *S. aureus* and *E. coli*, were quantitatively modeled using second-order polynomial equations Equations (4)–(8).

**Figure 1:**
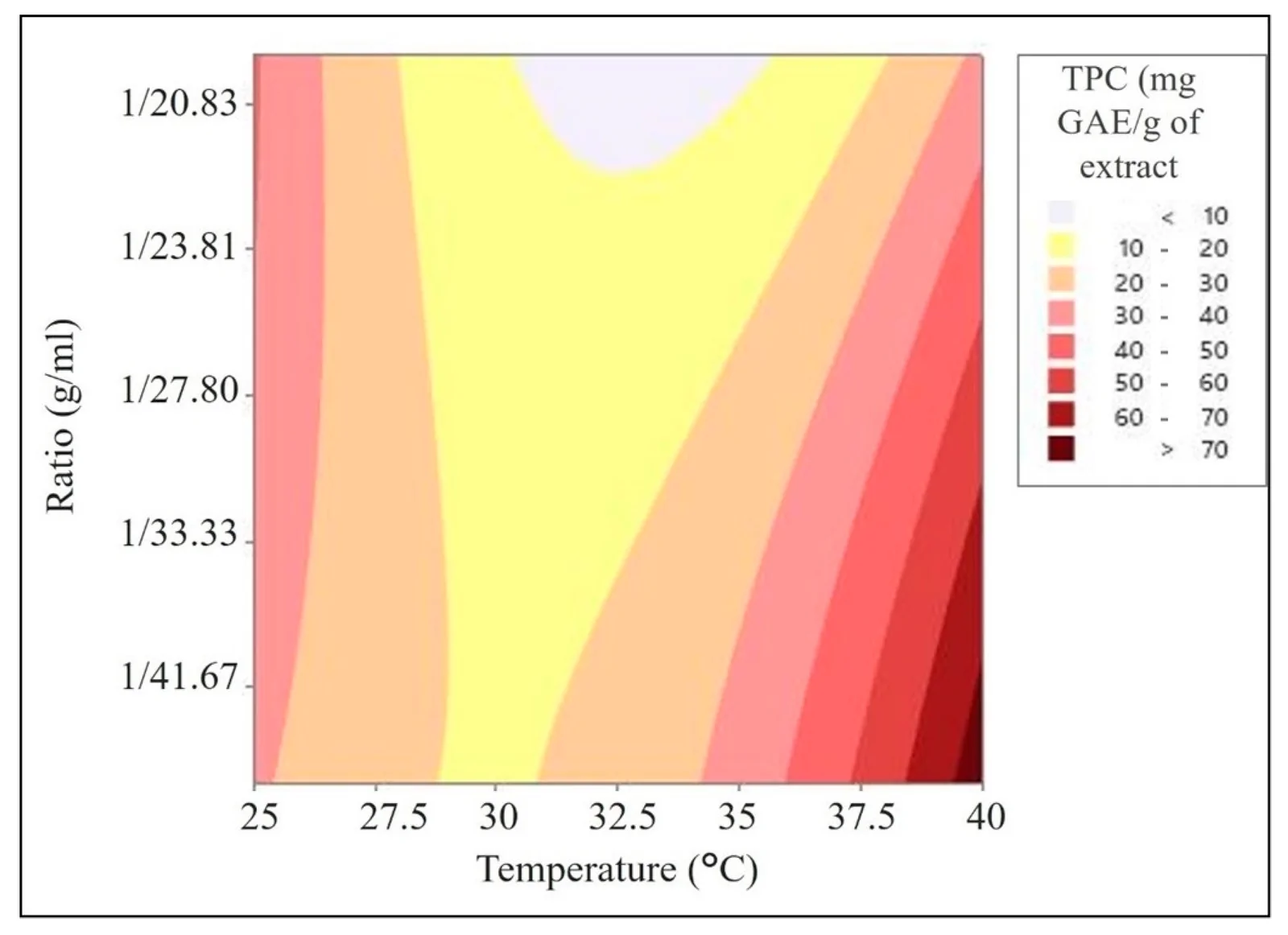
Contour plots showing the effect of ratio and temperature on total phenolic content.

**Figure 2:**
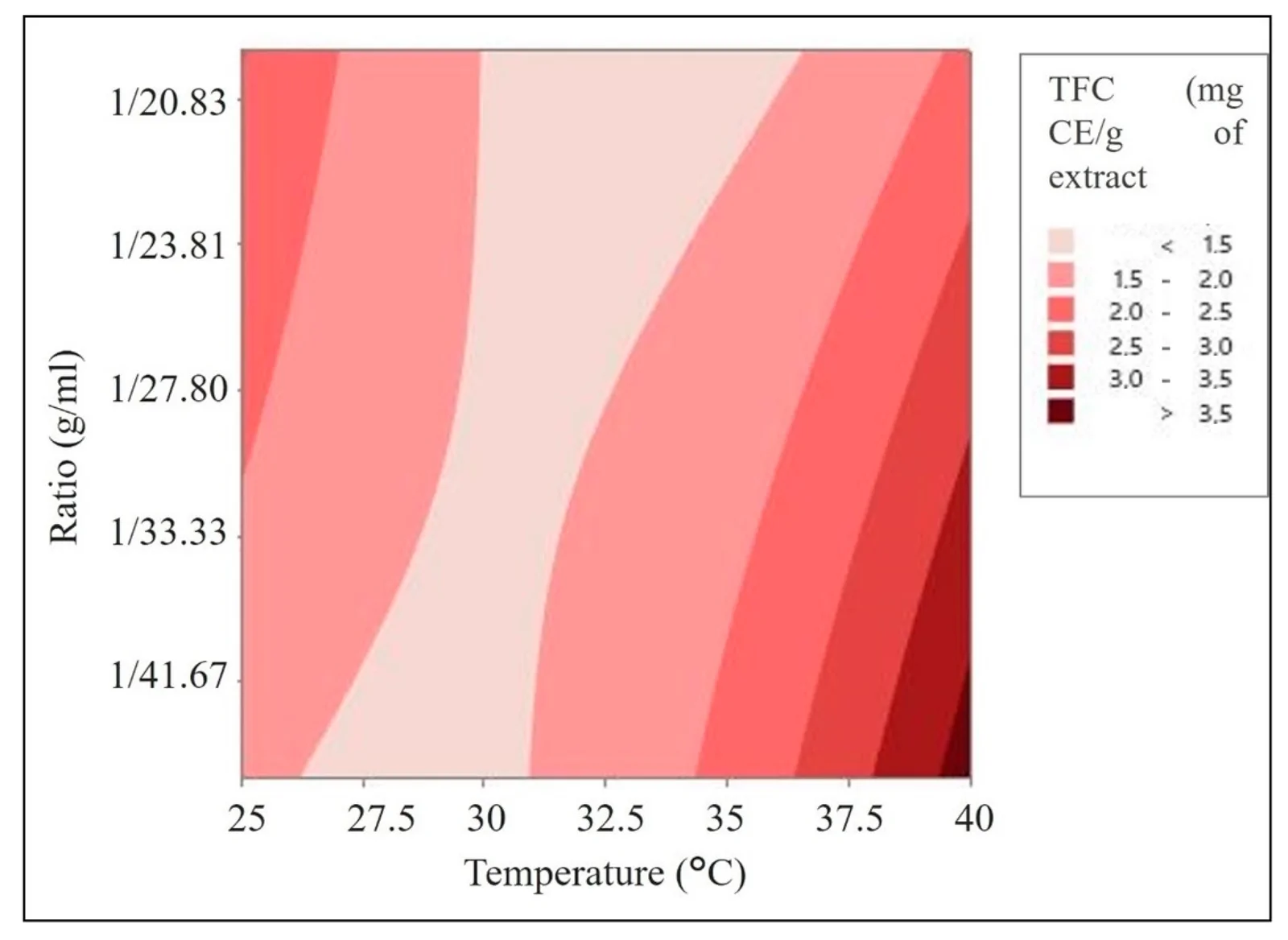
Contour plots showing the effect of ratio and temperature on total flavonoid content.

**Figure 3:**
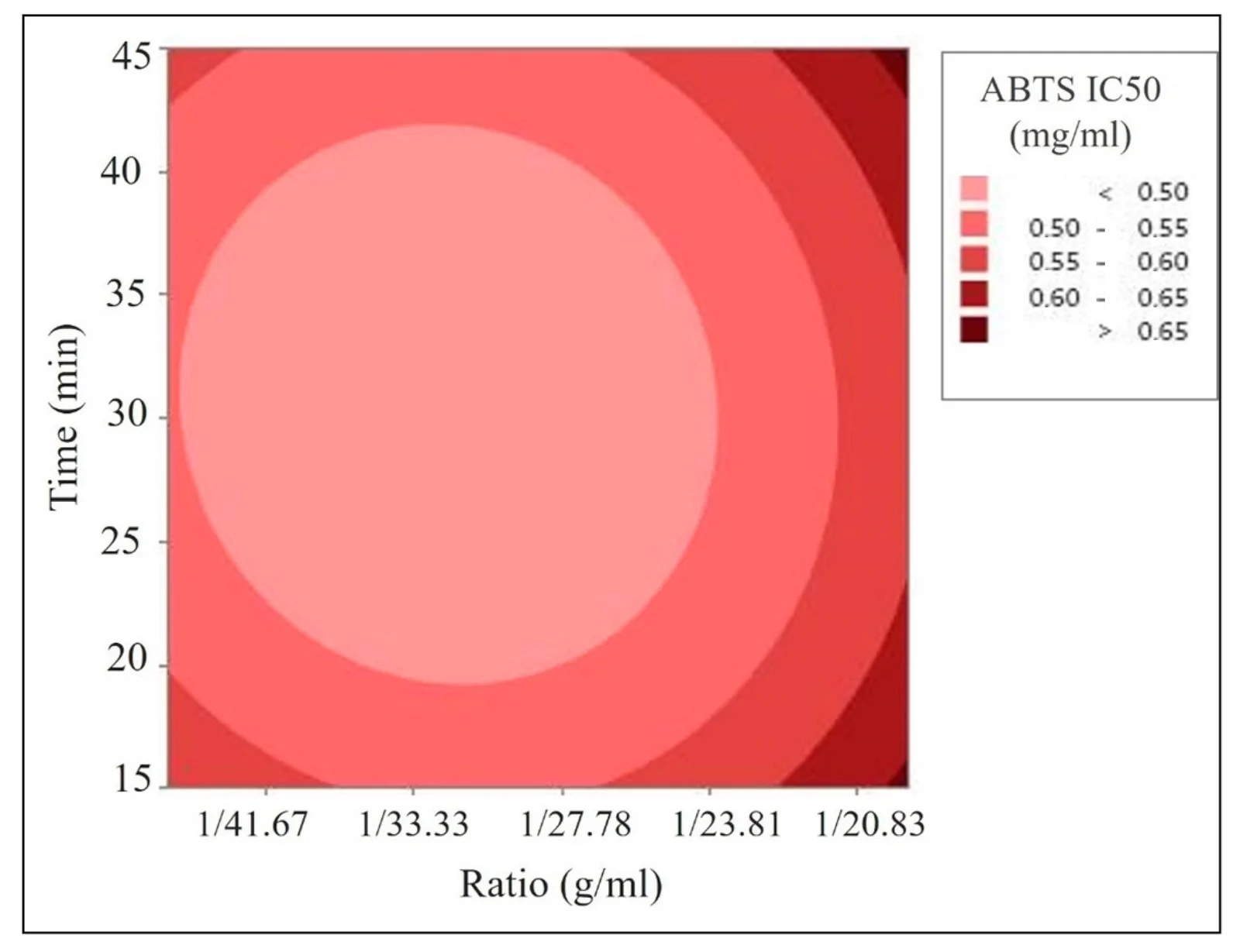
Contour plots showing the effect of ratio and time on ABTS scavenging activity.

**Figure 4:**
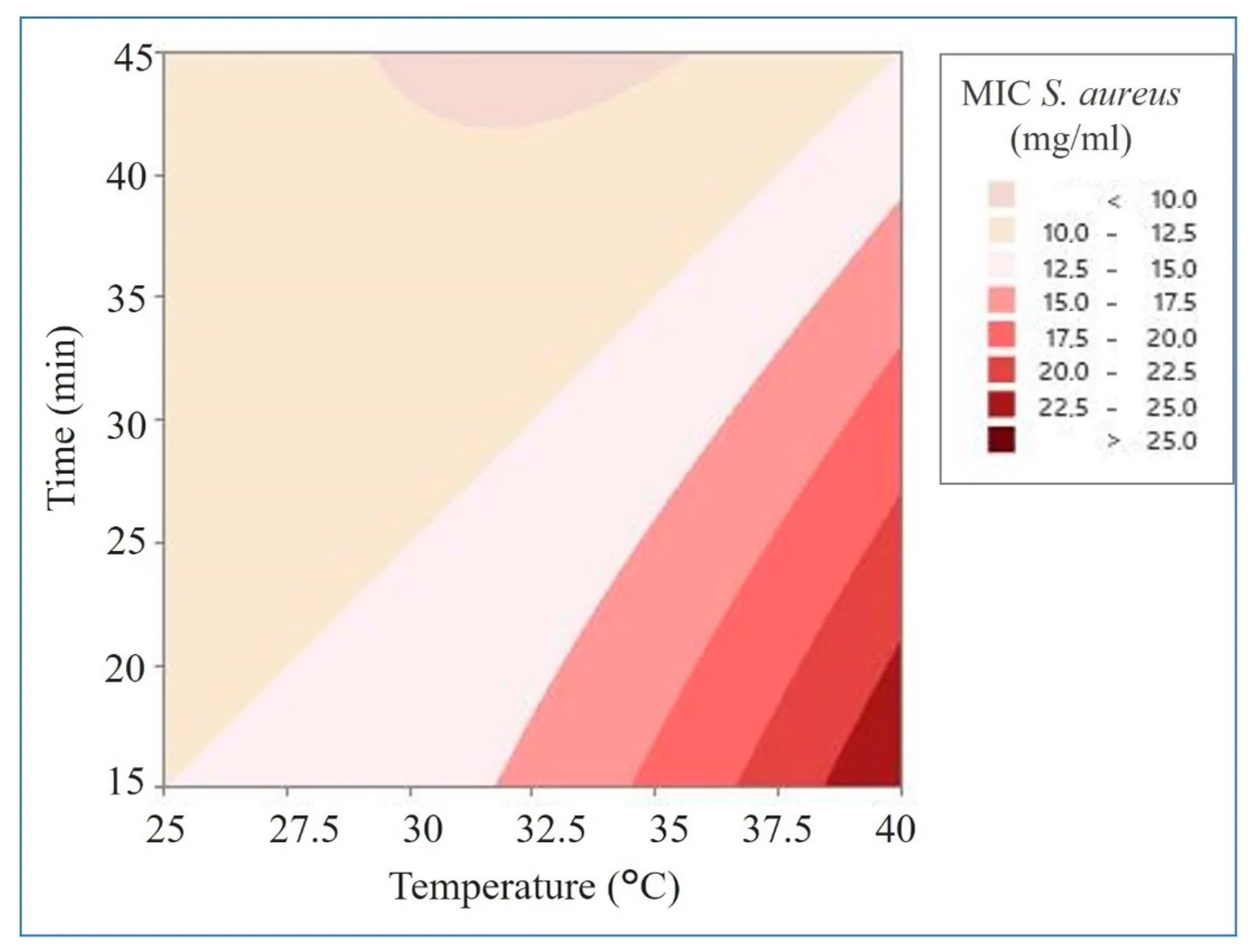
Contour plots showing the effect of time and temperature on anti-*S. aureus* activity.

**Figure 5:**
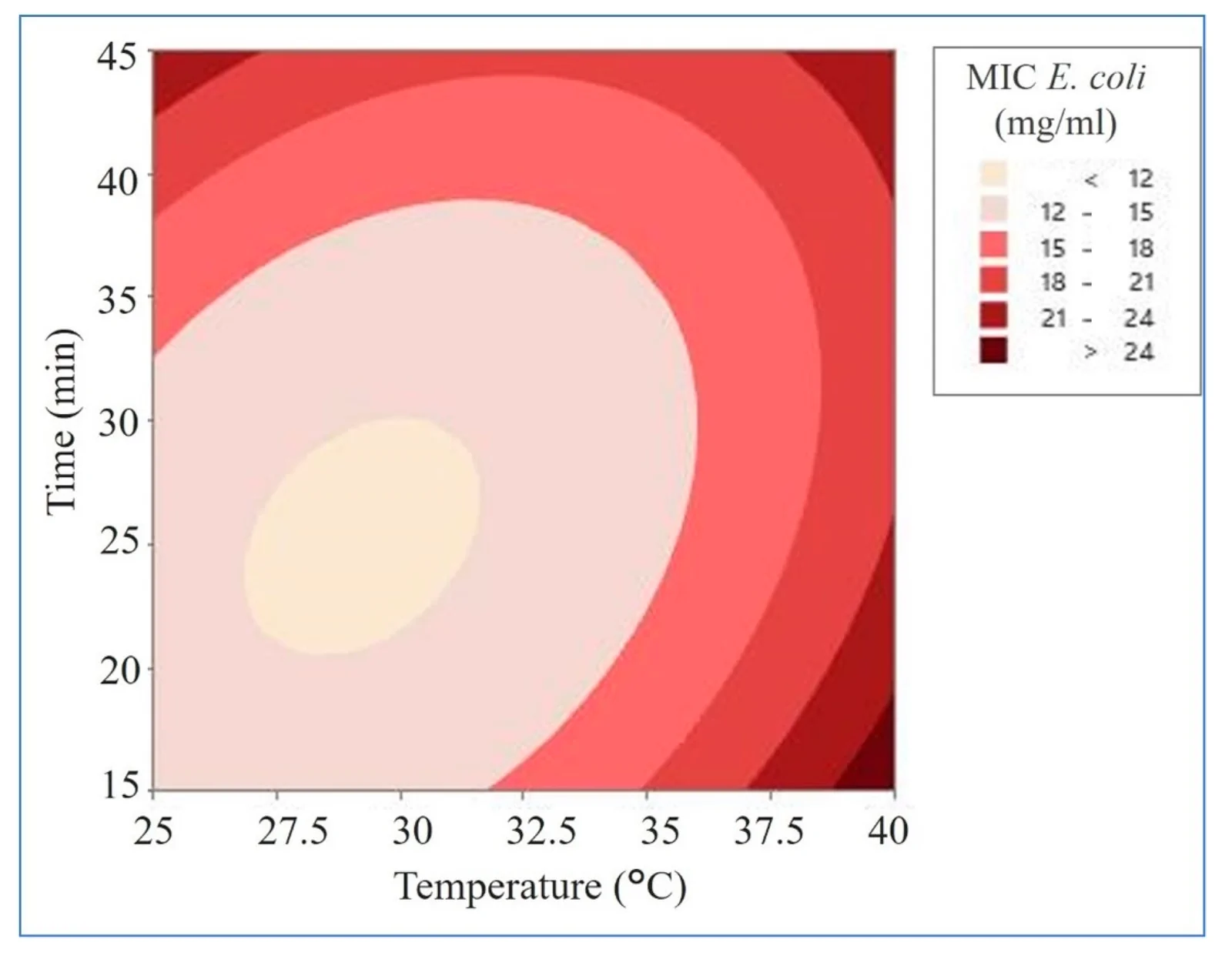
Contour plots showing the effect of time and temperature on anti-*E. coli* activity.

Furthermore, the lack-of-fit test of total phenolic content, total flavonoid content, and antioxidant capacity models was not significant (*p* > 0.05), indicating that the proposed equations were appropriate for predicting the response variables. The coefficients of determination (R^2^) were 99.89, 93.39, 97.57, 95.83 and 93.59% for total phenolic content, total flavonoid content, antioxidant capacity (ABTS radical scavenging), as well as antibacterial activity against *S. aureus* and *E. coli,* respectively, showing that approximately 90% of the experimental variability was explained by the models. These high values confirm that the models accurately represent the relationships among the studied variables. In addition, the adjusted coefficients of determination (R^2^ adj) for all the tested responses exceeded the acceptable threshold (>75%), further supporting model adequacy. Accordingly, the response data were fitted to quadratic polynomial regression models, retaining only statistically significant terms for predictive purposes. The interactions among X_1_, X_2_, and X_3_ and their effects on the responses were further visualized using two-dimensional contour plots, as displayed in Figure 1, Figure 2, Figure 3, Figure 4 and Figure 5.

### 3.1. Effect of Factors on Total Phenolic Content Extraction

Under the extraction conditions of a sample-to-solvent ratio of 1:50, a temperature of 40 °C, and a duration of 30 min, the maximum TPC was obtained in the whole fruit of *O. stricta* extract, which is 77.330 mg GAE/g extract (Table 2). This relatively high value can be explained by water’s efficiency as a polar solvent at controlled temperatures, which facilitates the extraction of hydrophilic phenolic compounds. In addition, the use of the whole fruit (including peel, pulp, and seeds) contributes to the higher phenolic yield, since the peel and seeds are known to contain substantially higher concentrations of bioactive compounds compared to the pulp alone. In contrast, Affi et al. [18] reported a lower TPC value (56 mg GAE/g extract) for the ethanolic extract of fruit peel obtained by maceration in ethanol for 24 h. Although ethanol is an efficient solvent for polyphenol extraction, the difference in yield compared to the aqueous whole-fruit extract can be attributed to the use of only the peel fraction and the long maceration at room temperature, which may lead to partial degradation or oxidation of phenolic compounds. A much lower TPC was observed in the study of El Mannoubi [34], where deseeded *O. stricta* fruit extracts presented values of 29.223 µg GAE/g in 80% acetone, 17.511 µg GAE/g in 80% methanol, and 16.349 µg GAE/g in 80% ethanol. The relatively low values in this case can be explained by the removal of seeds (which are a rich source of phenolics), as well as differences in extraction scale and solvent concentration.

The statistical results summarized in Table 3 indicate that, at the linear level, all three variables (X_1_, X_2_ and X_3_) significantly affected TPC: temperature, time, and ratio each had *p*-values below 0.01. In fact, the magnitude of the F-values revealed that temperature (X_1_) and sample-to-solvent ratio (X_3_) were the most influential linear factors, with F-values of 508.49 and 482.04, respectively, whereas extraction time (X_2_) exhibited a lower although still significant effect (F = 31.97).

The quadratic effects were even more pronounced than the linear effects. The quadratic term of temperature (X_1_^2^) indicated the highest F-value in the model (F = 2848.09), indicating that TPC was highly sensitive to temperature variations. Excessively high temperatures may lead to degradation, oxidation, or structural modification of thermolabile phenolic compounds. Regarding interaction effects, the interaction between temperature and sample-to-solvent ratio (X_1_ × X_3_) was the most influential interaction term (F = 572.39). This result highlights a strong synergistic relationship between these variables, suggesting that the effect of temperature on phenolic extraction depended on the level of solvent available and vice-versa. The interaction between temperature and time (X_1_ × X_2_) was also significant (F = 59.63), indicating that extraction efficiency was influenced by the combined effect of thermal energy and extraction duration. In contrast, the interaction between time and sample-to-solvent ratio (X_2_ × X_3_) depicted the lowest F-value (F = 14.10), suggesting a comparatively weaker combined effect. The results demonstrate that temperature emerged as the most influential parameter affecting phenolic extraction, followed by the sample-to-solvent ratio, while extraction time exhibited a comparatively lower contribution to the extraction efficiency.

The proposed second-order polynomial model (Equation (4)) describes the corresponding regression behavior:

Y TPC (mg GAE/g) = 406.1 − 29.143 × X_1_ + 0.042 × X_2_ + 4127 × X_3_
 + 0.5475 × X_1_^2^ + 0.02778 × X_2_^2^ − 8442 × X_3_^2^ − 0.03806 × X_1_X_2_ − 117.92 × X_1_X_3_ (4)

The contour plot (Figure 1) revealed that the highest TPC was obtained at moderate temperature (up to 40 °C) and a low ratio between 1/41.67 and 1/50, indicating that moderate heating likely improved phenolic release from the complex whole-fruit matrix. Similar behavior was depicted by Zeghbib et al. [35] when they evaluated TPC recovery from *Opuntia ficus* indica.

### 3.2. Effect of Factors on Total Flavonoids Content Extraction

The total flavonoid content (TFC) of the whole fruit of *O. stricta* extract was experimentally obtained and ranged from 1.182 to 3.910 mg CE/g extract, while the predicted values ranged from 1.30 to 3.78 mg CE/g extract. The strong similarity between the experimental values and the predicted results indicates that the developed model successfully explained the influence of extraction parameters on TFC. This agreement confirms the reliability and precision of the optimization model. The highest TFC value (3.910 mg CE/g extract) was obtained at a temperature of 40 °C, an extraction time of 30 min, and a ratio of 1:50. In contrast, the lowest TFC value (1.182 mg CE/g extract) was observed at 32.5 °C, 45 min, and a ratio of 1:20. These findings suggest that moderate-to-high temperatures combined with lower solvent ratios enhance the extraction of flavonoids.

The obtained TFC values of the OS extract reached 3.910 mg CE/g extract under 40 °C, a solvent-to-sample ratio of 1:50, and 30 min extraction, reflecting the overall flavonoid contribution of pulp, seeds, and peel combined. In comparison, Affi et al. [18] reported a higher value equal to 24.6 (mg CE/g) for the ethanolic extract of *O. stricta* fruit. This difference can be mainly attributed to the longer extraction time and ethanol maceration process, which enhance solvent penetration and compound diffusion.

The statistical results summarized in Table 4 indicate that at the linear level, only the temperature (X_1_) significantly affected the TFC value. With *p*-values = 0.014 (*p* < 0.05). In addition, the quadratic term (X_1_^2^) and the interaction term X_1_ × X_3_ (Temperature× Ratio) presented a significant effect (*p* < 0.05) on TFC content. The corresponding regression behavior was described by the proposed second-order polynomial model Equation (5):

Y TFC (mg CE/g) = 9.35 − 0.846 × X_1_ + 0.01805 × X_1_^2^ − 5.64 × X_1_X_3_ (5)

The response contour plot indicated that the highest TFC (>3.5 mg CE/g) was achieved at higher temperatures (40 °C), combined with a lower sample-to-solvent ratio (Figure 2). This suggests that moderate heating enhanced the release of flavonoids from the OS fruit matrix, improving their extraction efficiency.

### 3.3. Effect of Factors on Antioxidant Activities

The antioxidant activity of the different extracts evaluated by the ABTS assay presented IC_50_ values ranging from 0.188 to 0.696 mg/mL, while the positive control (ascorbic acid) depicted an IC_50_ value of 0.103 mg/mL, confirming its higher antioxidant capacity compared to the extracts (Table 2). Since lower IC_50_ values correspond to stronger radical scavenging activity, the results indicate considerable variations in antioxidant potential depending on the extraction conditions. Run 6, which corresponds to the highest antioxidant activity (ABTS scavenging activity), was obtained at 40 °C, 30 min, ratio 1:50, with the lowest IC_50_ value of 0.188 mg/mL. When compared to the results of Pino et al. [10], who reported an IC_50_ value of 13.24 mg/mL for the hydroalcoholic extract of *O. stricta* pulp obtained by maceration, our results demonstrate much stronger antioxidant activity. This large difference can be explained primarily by the use of the whole fruit instead of only the pulp, since the pulp is generally poorer in phenolic and flavonoid compounds than the peel and seed [10]. Compared to El Mannoubi [34], who reported ABTS IC_50_ values of 1.793 mg/mL (acetone), 3.768 mg/mL (methanol), and 4.074 mg/mL (ethanol) for deseeded *O. stricta* fruit extracts, our results still show a significantly higher antioxidant activity. This difference can be attributed to the inclusion of seeds and peel in our whole fruit extract, which are known to be richer in bioactive phenolic compounds, as well as to the use of water as an extraction solvent under optimized conditions, which may enhance the recovery of highly polar antioxidant compounds that contribute strongly to ABTS scavenging activity. Hence, the antioxidant activity of *OS* extract is highly dependent on both the analyzed plant part and the extraction methodology.

The statistical results summarized in Table 5 indicated that, at the linear level, the temperature (X_1_) and the ratio (X_3_) significantly affected ABTS scavenging activity, with *p*-values of 0.036 and 0.034, respectively. In addition, the quadratic terms (X_1_^2^, X_2_^2^ and X_3_^2^) and the interaction terms (X_1_ × X_2_, temperature × time) and (X_1_ × X_3_, temperature × ratio) exerted a significant effect (*p* < 0.05) on ABTS scavenging activity. The corresponding regression behavior was described by the following Equation (6):

Y ABTS (IC_50_ mg/mL) = −1.157 + 0.1982 × X_1_ − 52.02 × X_3_ − 0.003863 × X_1_^2^ + 0.0003565 × X_2_^2^ + 384.3 × X_3_^2^ + 0.000624 × X_1_X_2_ + 0.822 × X_1_X_3_ (6)

The contour plot illustrates the combined effect of extraction time and solvent ratio on the ABTS IC_50_ values of the OS extract (Figure 3). The lightest pink region in the center of the graph corresponds to the lowest IC_50_ values (<0.50 mg/mL), indicating the highest antioxidant activity. These favorable results were mainly obtained at moderate extraction times, between about 25 and 35 min, with medium solvent ratios ranging from 1/33.33 to 1/27.78 g/mL. The findings show that moderate extraction conditions lead to better recovery of antioxidant compounds.

### 3.4. Effect of Factors on Antibacterial Activities

As indicated in Table 2, the OS extract demonstrated inhibitory activity against both tested bacterial strains, *S. aureus* and *E. coli*, with MIC values ranging from 6.25 to 25 mg/mL. In fact, lower MIC values indicate stronger antibacterial activity. For *S. aureus*, the lowest MIC value (6.25 mg/mL), corresponding to the highest antibacterial activity, was obtained at Runs 6, 7, 10 and 12. Similarly, the antibacterial activity against *E. coli* reached a MIC value of 6.25 mg/mL at Runs 6 and 7. When compared with the study of Affi et al. [18], who reported lower MIC values for the ethanolic peel extract of *O. stricta*—namely, 1.68 mg/mL against *S. aureus* and 2.34 mg/mL against *E. coli*—our results present a weaker antibacterial potency. This difference can be mainly explained by the use of ethanol as an extraction solvent in their study, which may favor the extraction of more lipophilic antimicrobial compounds.

Regarding the analysis of variance of anti-*S. aureus* activity, Table 6 reveals that at the linear level, temperature and time significantly affected the activity. X_1_ and X_2_ each had *p*-values < 0.05 for both. In addition, the quadratic terms (X_1_^2^ and X_3_^2^) and the interaction terms X_1_ × X_2_ = (Temperature × time), X_1_ × X_3_ = (Temperature × Ratio) exhibited a significant effect (*p* < 0.05) on anti-*S. aureus* activity. These relationships are mathematically expressed in the corresponding regression model (Equation (7)):

Y MIC *S. aureus* (mg/mL) = 79.3 − 4.31 × X_1_ + 0.694 × X_2_
 + 0.0556 × X_1_^2^ − 13889 × X_3_^2^ − 0.02778 × X_1_X_2_ + 55.56 × X_1_X_3_ (7)

The contour plot (Figure 4) shows the combined effect of extraction temperature and time on the MIC against *S. aureus*. The lightest region corresponds to the lowest MIC values (<10 mg/mL), which means a high antibacterial effect. The maximum activity appears mainly at lower to moderate temperatures, around 27–32 °C, combined with longer extraction times of 40–45 min. The results suggest that moderate heating with sufficient extraction time improves the release of active antibacterial compounds.

The analysis of variance of anti-*E. coli* activity revealed that the linear factor, temperature (X_1_), had a significant influence on the antibacterial effect (Table 7). The quadratic terms (X_1_^2^, X_2_^2^, and X_3_^2^) also showed significant effects, indicating that variations in these parameters strongly affected the antibacterial response. Among the interaction effects, the interaction between temperature and ratio (X_1_ × X_3_) was found to be statistically significant (*p* < 0.05), demonstrating that the combined influence of these two factors played an important role in the antibacterial activity. Based on these results, a second-order polynomial Equation (8) was developed to predict the relationship between the experimental variables and the anti-*E. coli* activity:

Y MIC *E. coli* (mg/mL) = 109.5 − 6.11 × X1 + 0.0833 × X12 + 0.02083 × X22 − 20,833 × X32 + 55.6 × X1X3 (8)

The contour plot illustrates the effect of extraction temperature and extraction time on the MIC against *E. coli*. The light-colored region located around 31 °C and 28 min corresponds to the lowest MIC values (<12 mg/mL). This indicates that the extract exhibited the highest antibacterial activity under moderate extraction conditions. As the temperature and extraction time increased beyond these moderate levels, the MIC values gradually increased. This means that the antibacterial activity decreased at higher temperatures and longer extraction times. In line with these findings, hydroethanolic extracts derived from the whole fruit and the pulp of *Opuntia ficus*-indica have demonstrated broad-spectrum antibacterial activity against *S. aureus, Salmonella typhi,* and *E. coli* [36]. In fact, the antibacterial effect may be linked to the interaction of bioactive compounds present in the OS extract with the microbial cell membrane, which can modify its structure, eventually resulting in cell damage and disruption [37].

### 3.5. Optimization and Experimental Validation

**Figure 6:**
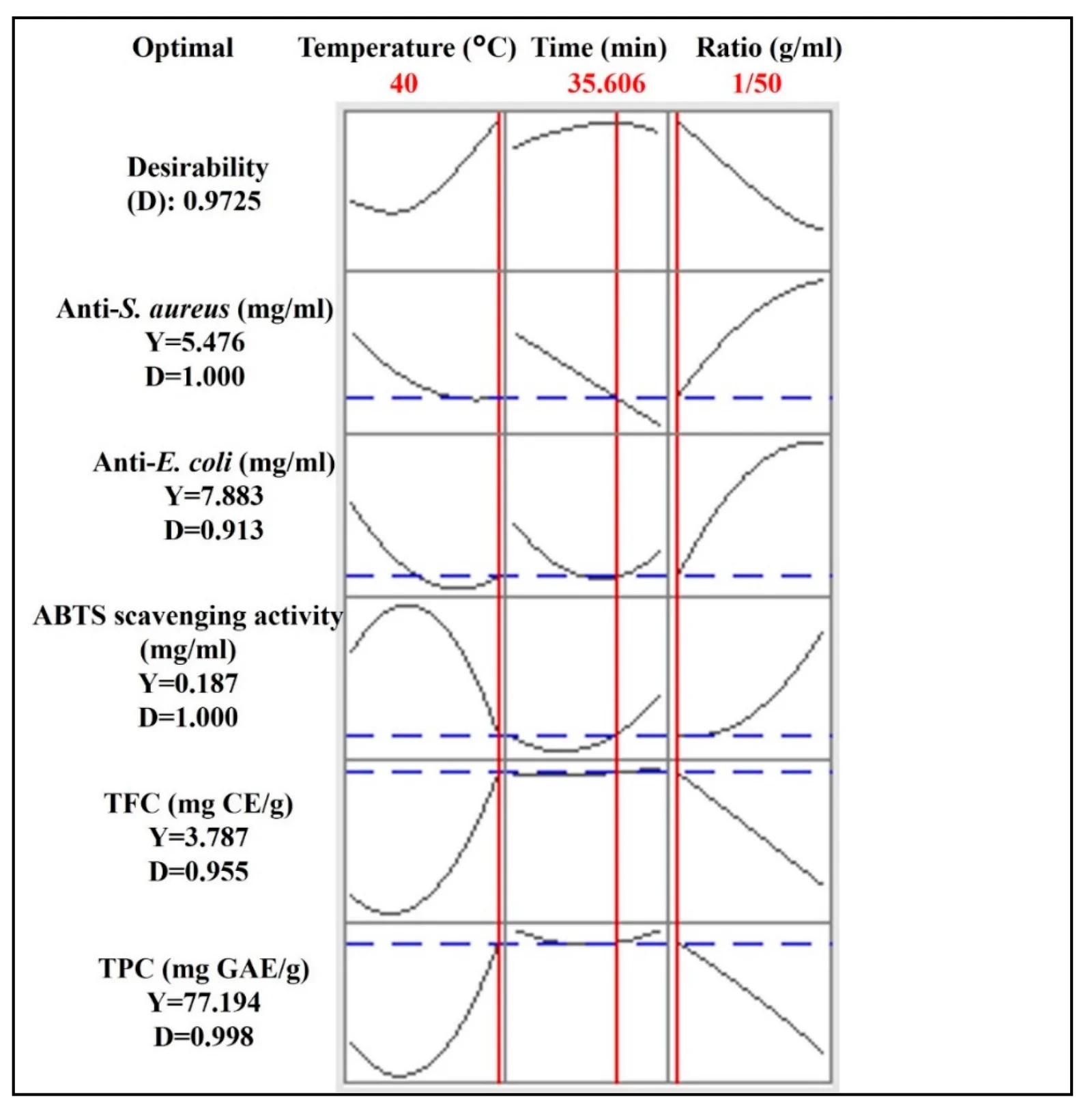
Response optimizer under the optimum conditions for the maximum response of TPC, TFC and ABTS scavenging activity, as well as anti-*S. aureus* and anti-*E. coli* activities.

A numerical optimization approach was applied to determine the most suitable conditions for the different extracts. The aim was to identify the optimal levels of factors, including extraction temperature, extraction time, and sample-to-solvent ratio, to maximize all responses simultaneously. The optimized extraction conditions were a temperature of 40 °C, an extraction time of 35.6 min and a sample-to-solvent ratio of 1:50 (Figure 6). These conditions were considered optimal based on the RSM optimization results, with an overall desirability value of 0.9725. Under these optimized conditions, the predicted maximum values for TPC, TFC, ABTS scavenging activity and anti-*S. aureus*, and anti-*E. coli* activities were 77.194 mg GAE/g, 3.787 mg CE/g, 0.187 mg/mL, 5.476 mg/mL and 7.883 mg/mL, respectively.

In addition, to validate the model, an additional experiment was performed under the predicted optimal conditions (40 °C, 35.6 min, and a solid-to-liquid ratio of 1:50), and the experimental responses were compared with the model predictions. According to Table 8, responses TPC, TFC, and ABTS radical-scavenging activity and anti-*S. aureus* activity show relatively good agreement between predicted and experimental values, with relative errors ranging from 0.05% to 9.52%. However, anti-*E. coli* activity exhibits a considerably larger deviation (21.17%), indicating that the model was less accurate at predicting this response under optimal conditions. This limitation may be partly attributed to the discrete nature of MIC measurements, which are generally determined using predefined concentration levels and may therefore provide fewer continuous response values for fitting a polynomial model.

### 3.6. Principal Component Analysis (PCA)

**Figure 7:**
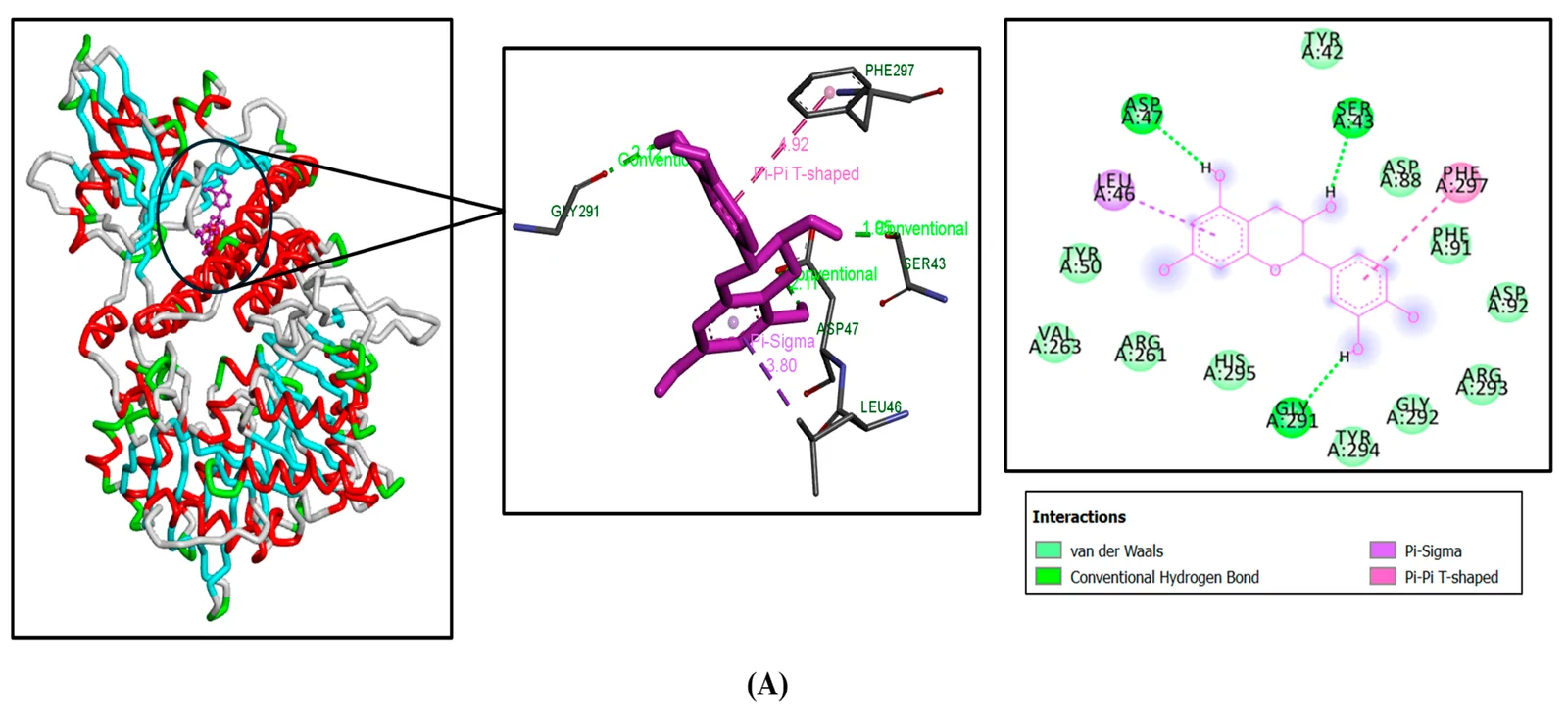

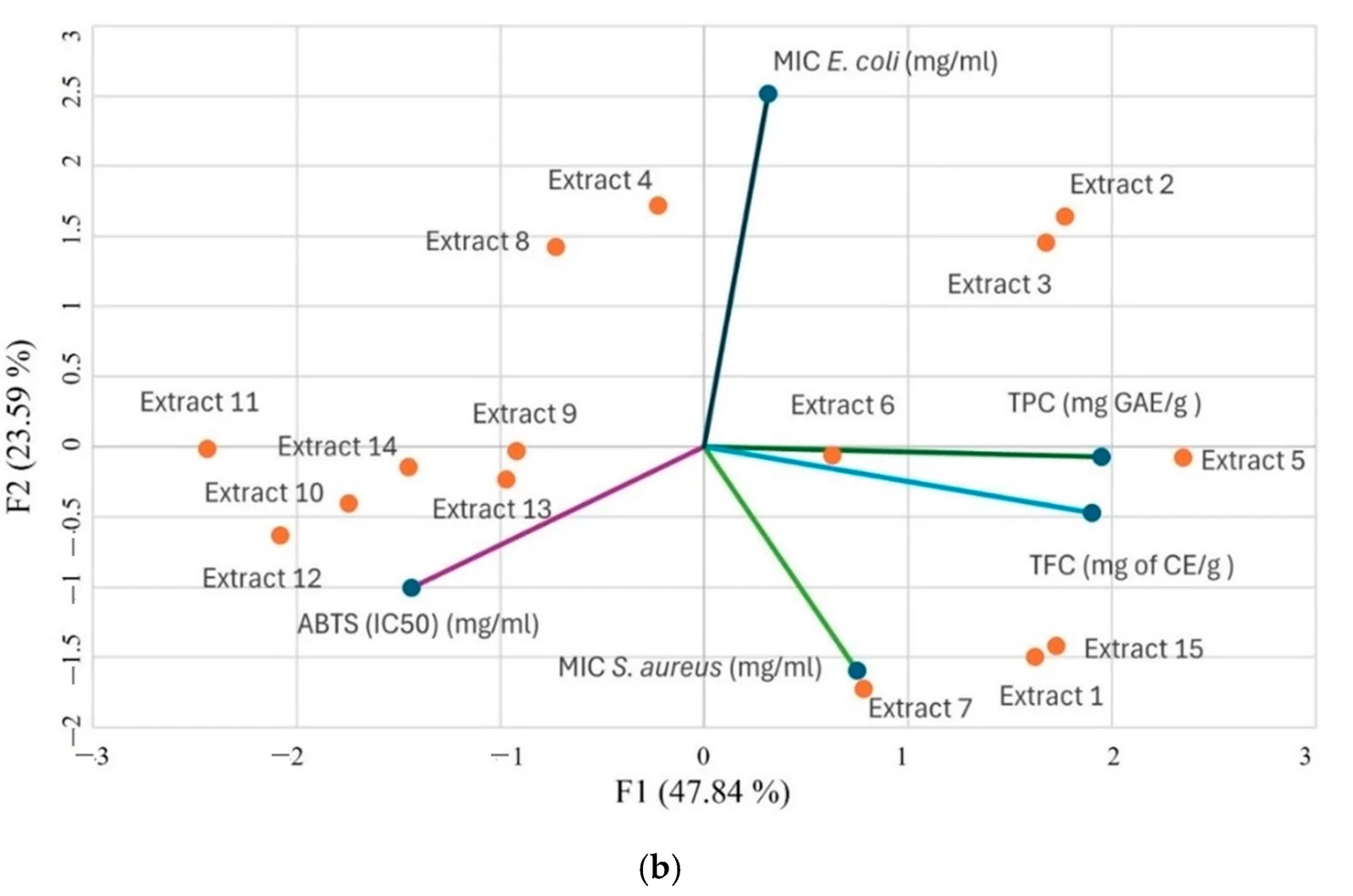
Principal component analysis (PCA) plots showing (**a**) the distribution of phytochemical parameters, including TPC and TFC, together with biological activities, and (**b**) the distribution of all studied extracts (1–15).

With the purpose of revealing hidden patterns and similarities between the different OS extracts, phytochemical contents and their bioactivities were investigated. The dataset obtained was studied using principal component analysis (PCA). PCA generates score plots of the distribution of the different samples following the major components. In this study, the obtained score plots (Figure 7) highlighted the differences between the different OS extracts, phytochemical contents and their bioactivities. The major principal component is F1, which contributed to 47.84% of the total variance. In fact, F1 has a maximum eigenvalue of 2.39, while F2, F3, F4, and F5 have eigenvalues of 1.179, 0.828, 0.565 and 0.030 and elucidate 23.59, 16.56, 11.3 and 0.708% of the variance in the data, respectively. These results show that the model provided a satisfactory description of the relationships between the studied extracts and variables. Consequently, and based on the loading plot of PCA, F1 and F2, which contributed to 71.43% of the total variance, were utilized to supply a relevant visual guide for mentioning data differences (Figure 7). Indeed, the F1 axis is associated with the majority of variables (TPC, TFC and ABTS) with high values, whereas the F2 axis is linked to MIC of *S. aureus* and MIC of *E. coli*)

The projection of variables revealed that TPC and TFC were grouped closely together on the positive side of F1, indicating a strong positive relationship between these parameters. This trend is consistent with previous reports [38,39], which also found a significant correlation between TPC and TFC. On the other hand, ABTS values were located on the opposite side of the axis, showing a negative correlation with phenolic compounds. This means that extracts with higher phenolic levels exhibited stronger antioxidant activity, as reflected by lower IC_50_ values. A similar behavior was observed by El Mannoubi [34,40], who demonstrated a significant negative correlation between phenolic compounds and ABTS activity.

The MIC values against *S. aureus* appeared relatively close to the phenolic variables, suggesting that phenolic compounds may play an important role in the antibacterial activity against Gram-positive bacteria. In contrast, the MIC values for *E. coli* were separated along F2, indicating a different behavior and a weaker association with phenolic composition. This difference may be explained by the higher resistance of Gram-negative bacteria due to the presence of an outer membrane. Concerning the extracts, extracts 2 and 3 were positioned near TPC and TFC, highlighting their richness in bioactive compounds and their strong biological potential (Figure 7). In comparison, extracts 10, 11, 12, and 13 were located closer to the ABTS variable, suggesting lower antioxidant efficiency.

### 3.7. Quantification of the Optimized Whole Fruit Extract of Opuntia Stricta

**Figure 8:**
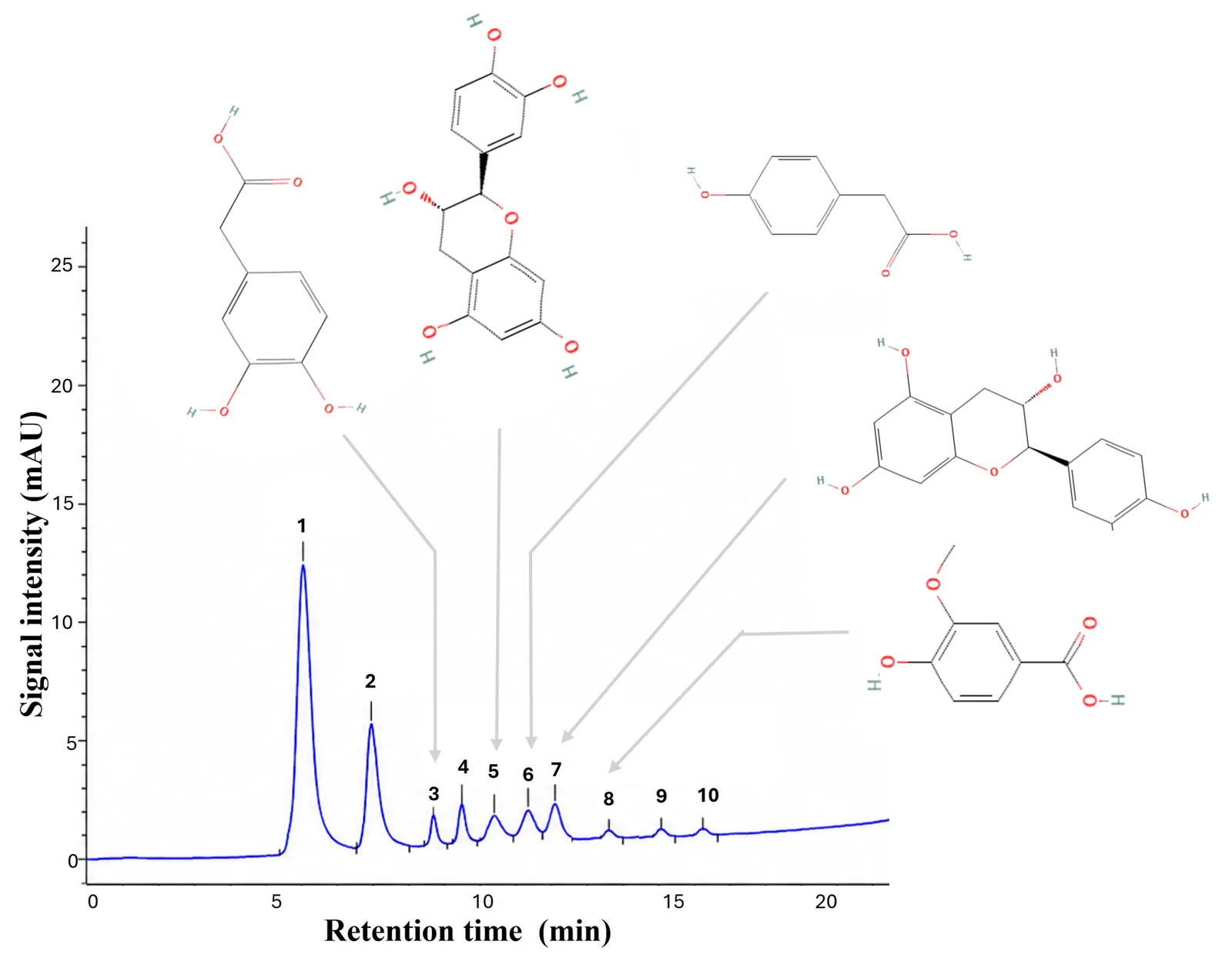
HPLC-DAD chromatogram illustrating the phenolic profile of the optimized *Opuntia stricta* extract detected at 280 nm.

The qualitative and quantitative analysis of phenolic compounds in aqueous extracts of the whole fruit extract under optimal conditions was determined by High-Performance Liquid Chromatography (HPLC). The HPLC analysis revealed the presence of ten compounds, five of which were successfully identified, including two flavonoids and three phenolic acids (Table 9). Among the identified phenolic acids were 4-hydroxyphenylacetic acid (HPAC), vanillic acid and 3,4-dihydroxyphenylacetic acid (DOHPAC). HPAC was the most abundant, with a value of 14.86 mg/g (Figure 8). Among the identified flavonoid compounds, only catechin and epicatechin were detected in the OS extract, with concentrations of 0.43 to 6.05 mg/g, respectively.

The catechin concentration identified in the present study was 0.43 mg/g. These values were considerably higher than those reported by Loukili et al. [41] for ethanolic extracts of *Opuntia dillenii* peels, in which catechin was found at a concentration of 0.323 mg/g. Similarly, in another study, Loukili et al. [21] reported lower levels of phenolic compounds in aqueous extracts of *O. dillenii* juice, with vanillic acid and catechin contents of 0.044 mg/g and 0.123 mg/g, respectively. In the same study, aqueous peel extracts contained 0.507 mg/g vanillic acid, a value that was comparable to those obtained in the present investigation. The observed differences may be related to several factors, including environmental growing conditions, fruit maturity and parts, and extraction procedures.

### 3.8. In Silico Simulation of the Antibacterial Potential of Opuntia Stricta Phytochemicals

In this section, a molecular docking approach was applied to investigate the antibacterial potential of *Opuntia stricta* compounds. Two main targets were selected: the PPK of *E. coli* and the Fibronectin binding protein A (FnBPA) of *S. aureus*. It is important to mention that the polyphosphate kinase (PPK) is a crucial enzyme involved in the synthesis of inorganic polyphosphate and the regulation of multiple bacterial physiological processes [42]. It participates in the metabolism of bacterial inorganic polyphosphate (polyP) and catalyzes the formation of polyP from ATP in a reversible way. The latter contributes significantly to bacterial pathogenicity and resistance by stimulating biofilm formation, regulating energy metabolism, improving motility, reinforcing stress-response mechanisms, and ensuring bacterial persistence [43]. On the other hand, fibronectin-binding protein A is a cell wall adhesin that mediates high-affinity binding to host fibronectin, promoting colonization, invasion, immune system bypassing, and biofilm formation. Results of homology modeling displayed that the FnBPA model identity was 99%, with a Qualitative Model Energy Analysis (QMEAN) value equal to −0.37 and over >90% of residues were in favored regions and allowed regions of the Ramachandran Plot. These quality indicators confirm the reliability and relevance of the predicted model for subsequent simulation studies. Furthermore, molecular docking calculations revealed that the two compounds, catechin and epicatechin, showed the strongest free energies of binding towards both targets, with scores ranging from −7.9 kcal/mol to −8.2 kcal/mol, indicating a potential to interact with the selected targets (Table 10). Nevertheless, further experimental studies are still required to corroborate these computational predictions.

A previous study indicated that catechin showed promising inhibitory potential against the MurB enzyme involved in peptidoglycan synthesis [44]. Additionally, Jubair et al. [45] demonstrated that catechin isolated from Canarium patentinervium Miq. might hinder the expression of biofilm formation by binding to the AcrB domain of the E. coli AcrAB-TolC efflux pump. Moreover, epicatechin inhibited the transpeptidase activity of sortase A of S. aureus by hindering biofilm formation and bacterial adhesion [46]. Gao et al. [47] stated that epicatechin can be used as a preventive and protective antibacterial agent owing to its ability to impede the regulation of virulence genes.

**Figure 9:**
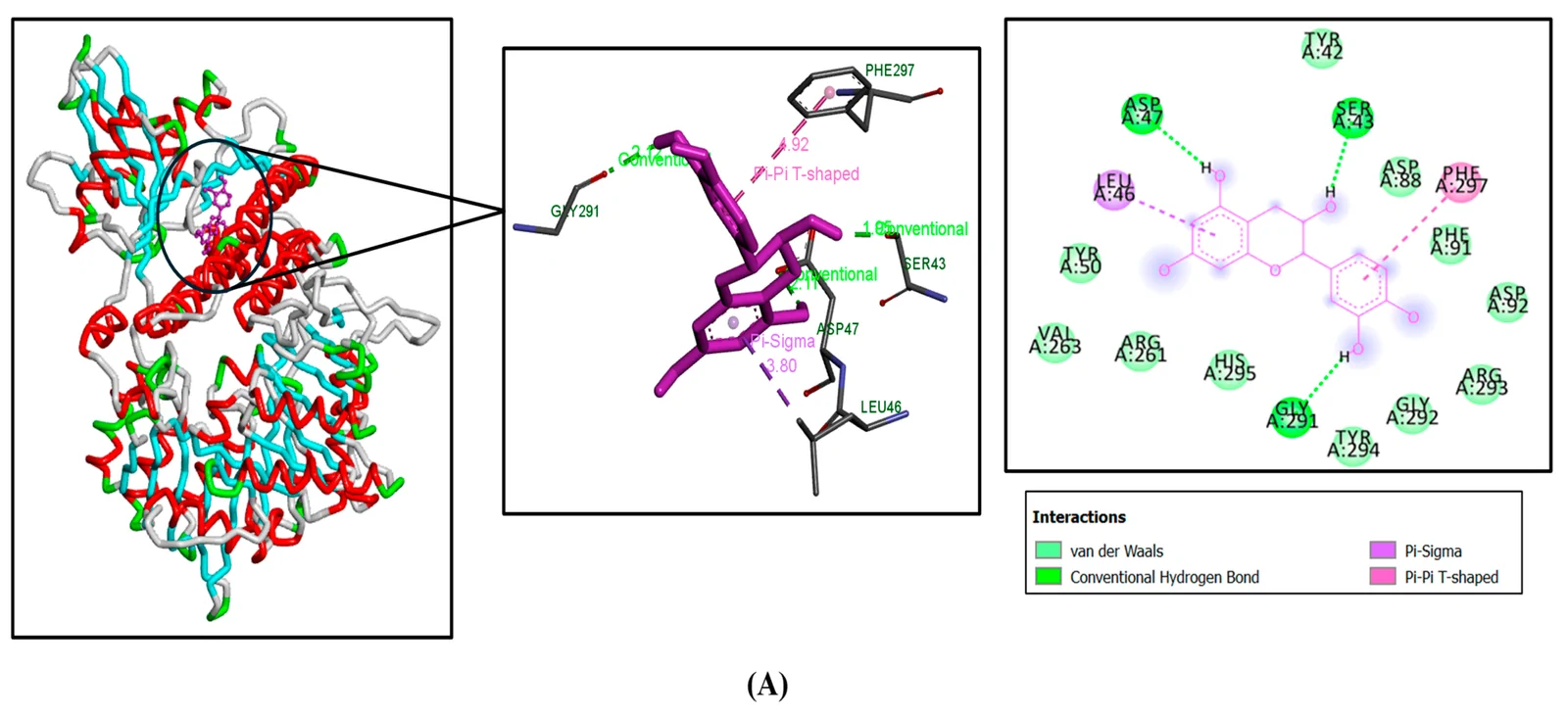

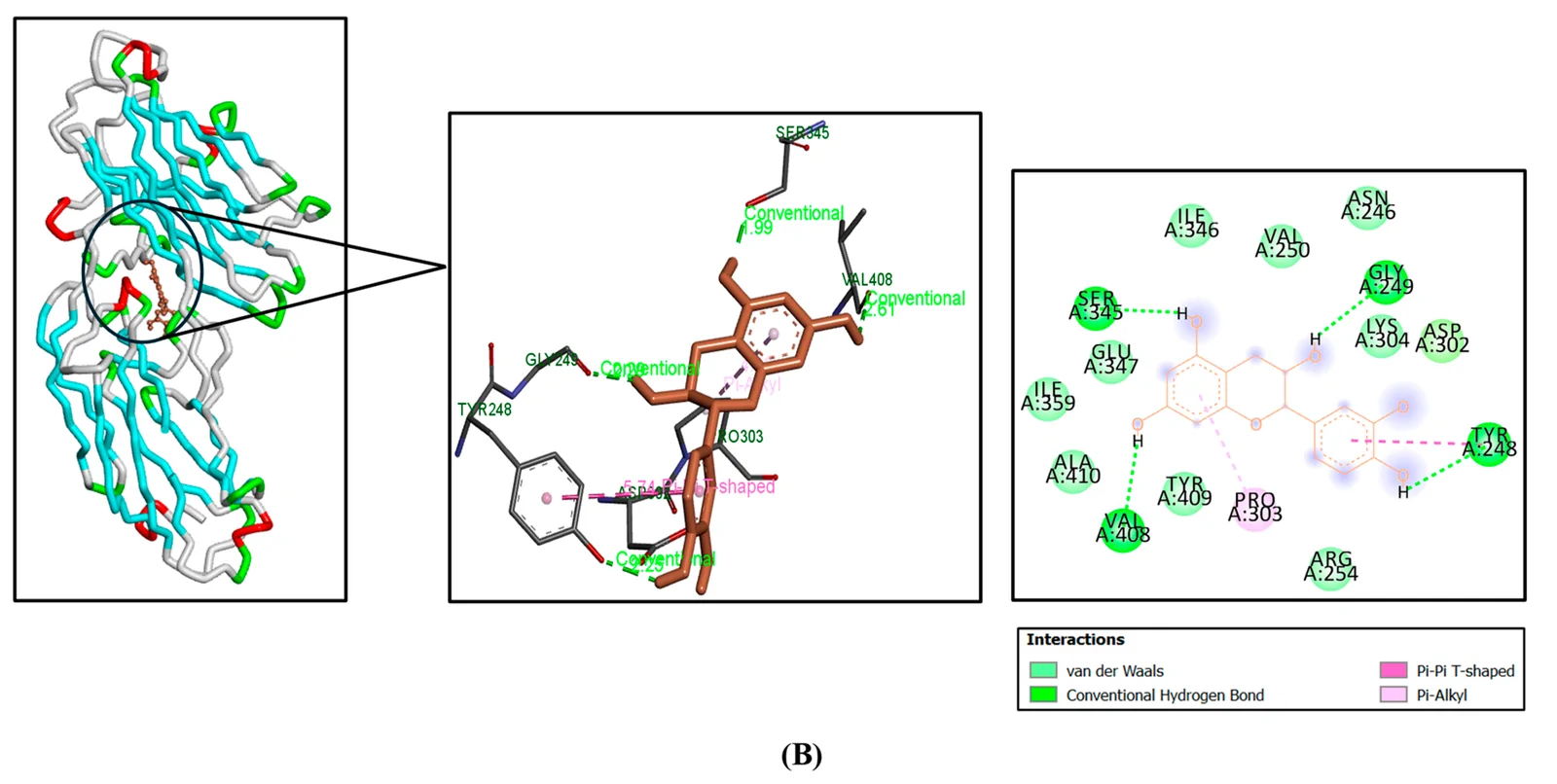
Interaction profiles of catechin against PPK of *E. coli* (**A**) and fibronectin-binding protein A of *S. aureus* (**B**).

The interaction profiles of catechin compounds, which showed the highest affinity against the PPK of *E. coli* and fibronectin-binding protein A (FnBPA) of *S. aureus*, are represented in Figure 9.

Catechin binds within the active pocket of *E. coli* PPK. It forms two hydrogen bonds with SER43 and ASP47 and shows distances ranging between 2.11 and 1.95 Å, respectively. Its aromatic ring makes a π–π T-shaped interaction with PHE297 and a π–sigma interaction Via LEU46, while GLY291 defines the pocket geometry (Figure 9A).

On the other side, it binds within fibronectin-binding protein A of *S. aureus* by forming multiple conventional hydrogen bonds with TYR248, GLY249, and SER345, with distances ranging between 1.99 and 2.74 Å. Its aromatic ring made π-alkyl and π-T-shaped interactions with hydrophobic residues such as PRO303 and VAL408 in order to ensure ligand stabilization (Figure 9B).

Therefore, these findings suggest that *Opuntia stricta extract compounds* may notably interact with the studied virulence factors. These preliminary results may support further In Vitro/In Vivo studies in order to tackle microbial pathogenicity and resistance to antibiotics. Moreover, Medicinal plant extracts may contain multiple bioactive compounds in varying abundances, and the overall activity of the extract is a result of the combined action of multiple compounds with synergistic or additive activities [48,49].

## 4. Conclusions

A Box–Behnken design was employed to optimize the extraction conditions based on the selected phytochemical and biological responses. Three factors were varied, and the response optimization approach allowed for the maximization of phytochemical content as well as antioxidant and antibacterial activities. In addition, principal component analysis allowed for a clear differentiation between the various OS extracts. PCA highlighted the relationship between phytochemical contents, particularly TPC and TFC, and the observed antioxidant and antibacterial activities through correlation analysis. The HPLC analysis of the optimized OS extract revealed that 4-hydroxyphenylacetic acid was the main phenolic acid present. Among the flavonoids, epicatechin was the most abundant compound. Furthermore, molecular docking simulations predicted that *Opuntia stricta* extract compounds, such as catechin and epicatechin, may exhibit significant interactions with bacterial virulence factors, including PPK of *E. coli* and fibronectin-binding protein A of *S. aureus*. Nevertheless, additional in vitro and in vivo studies are required to substantiate these computational predictions. Hence, the findings suggest that OSE possesses notable bioactive properties, highlighting its potential for further investigation in functional and industrial applications.

## Figures and Tables

**Table 1:** Experimental matrix.

Runs	X_1_: Temperature (°C)	X_2_: Time (min)	X_3_: Sample/Solvent Ratio (g/mL)
1	25	15	1:30
2	40	15	1:30
3	25	45	1:30
4	40	45	1:30
5	25	30	1:50
6	40	30	1:50
7	25	30	1:20
8	40	30	1:20
9	32.5	15	1:50
10	32.5	45	1:50
11	32.5	15	1:20
12	32.5	45	1:20
13	32.5	30	1:30
14	32.5	30	1:30
15	32.5	30	1:30

**Table 2:** Box–Behnken design variables for TPC, TFC, ABTS scavenging activity and anti-*S. aureus* and *E. coli* activity responses.

Runs	Factors			TPC (mg GAE/g Extract)		TFC (mg CE/g Extract)		ABTS (IC_50_) (mg/mL)		MIC *S. aureus* (mg/mL)		MIC *E. coli* (mg/mL)		Extraction Yield (%)
	X_1_: Temperature (°C)	X_2_: Time (min)	X_3_: Sample/solvent ratio (g/mL)	Exp.	Pred.	Exp.	Pred.	Exp.	Pred.	Exp.	Pred.	Exp.	Pred.	-
1	25	15	1:30	38.40 ± 0.40 ^f^	37.62	1.883 ± 0.011 ^g^	1.690	0.478 ± 0.001 ^d^	0.450	12.5 ^b^	12.86	12.5 ^b^	14.51	77.14 ± 0.51 ^d^
2	40	15	1:30	63.73 ± 0.191 ^b^	64.60	2.891 ± 0.009 ^b^	3.170	0.206 ± 0.001 ^i^	0.200	25 ± ^a^	24.86	25 ^a^	25.63	95.60 ± 0.70 ^b^
3	25	45	1:30	52.33 ± 0.157 ^d^	51.08	2.800 ± 0.008 ^c^	2.520	0.298 ± 0.001 ^g^	0.300	12.5 ^b^	12.36	25 ^a^	23.87	60.40 ± 0.66 ^g^
4	40	45	1:30	60.53 ± 0.182 ^c^	60.46	2.727 ± 0.008 ^d^	2.880	0.307 ± 0.001 ^g^	0.330	12.5 ^b^	12.34	25 ^a^	22.47	67.80 ± 0.66 ^f^
5	25	30	1:50	32.20 ± 0.097 ^h^	32.08	1.387 ± 0.004 ^i^	1.640	0.404 ± 0.001 ^f^	0.420	12.5 ^b^	13.90	12.5 ^b^	14.03	95.25 ± 0.58 ^b^
6	40	30	1:50	77.33 ± 0.232 ^a^	76.25	3.910 ± 0.012 ^a^	3.780	0.188 ± 0.001 ^j^	0.355	6.25 ^c^	7.656	6.25 ^c^	7.76	66.33 ± 0.61 ^f^
7	25	30	1:20	40.40 ± 0.121 ^e^	41.40	2.380 ± 0.007 ^e^	2.540	0.284 ± 0.001 ^h^	0.218	6.25 ^c^	5.562	6.25 ^c^	4.69	74.80 ± 0.66 ^e^
8	40	30	1:20	32.467 ± 0.097 ^gh^	32.50	2.364 ± 0.007 ^e^	2.140	0.438 ± 0.001 ^e^	0.422	25 ^a^	23.29	25 ^a^	23.44	94.13 ± 0.65 ^b^
9	32.5	15	1:50	25.267 ± 0.07 ^i^	25.32	1.515 ± 0.005 ^h^	1.420	0.573 ± 0.002 ^c^	0.660	12.5 ^b^	10.80	12.5 ^b^	9.35	98.00 ± 0.66 ^a^
10	32.5	45	1:50	32.933 ± 0.099 ^g^	33.92	2.042 ± 0.006 ^f^	2.070	0.580 ± 0.002 ^c^	0.670	6.25 ^c^	5.53	12.5 ^b^	12.45	95.25 ± 0.54 ^b^
11	32.5	15	1:20	13.33 ± 0.040 ^L^	12.26	1.463 ± 0.004 ^h^	1.460	0.655 ± 0.002 ^a^	0.663	12.5 ^b^	13.93	12.5 ^b^	12.50	93.60 ± 0.66 ^c^
12	32.5	45	1:20	12.667 ± 0.038 ^m^	12.54	1.182 ± 0.004 ^k^	1.300	0.696 ± 0.002 ^a^	0.672	6.25 ^c^	7.66	12.5 ^b^	15.62	98.00 ± 0.50 ^a^
13	32.5	30	1:30	16.333 ± 0.049 ^k^	17.59	1.364 ± 0.004 ^i^	1.540	0.463 ± 0.001 ^d^	0.460	12.5 ^b^	12.35	12.5 ^b^	12.25	92.50 ± 0.56 ^c^
14	32.5	30	1:30	17.031 ± 0.051 ^j^	17.59	1.861 ± 0.006 ^g^	1.540	0.472 ± 0.001 ^d^	0.460	12.5 ^b^	12.35	12.5 ^b^	12.25	92.00 ± 0.56 ^c^
15	32.5	30	1:30	16.733 ± 0.050 ^jk^	17.59	1.289 ± 0.004 ^j^	1.540	0.443 ± 0.001 ^e^	0.460	12.5 ^b^	12.35	12.5 ^b^	12.25	92.20 ± 0.45 ^c^

Exp: Experimental; Pred: Predicted; ±: Standard deviation of three replicates. Means followed by different letters (a–m) in the same column, for each parameter, are different (*p* < 0.05).

**Table 3:** Analysis of Variance (ANOVA) of total phenolic content.

Source	DF	Adjusted SS	Adjusted SM	F-Value	*p*-Value
Model	9	5687.73	631.97	513.88	0.000
Linear	3	1257.48	419.16	340.83	0.000
X1	1	625.34	625.34	508.49	0.000
X2	1	39.32	39.32	31.97	0.002
X3	1	592.82	592.82	482.04	0.000
Quadratic	3	3635.66	1211.89	985.43	0.000
X1 × X1	1	3502.59	3502.59	2848.09	0.000
X2 × X2	1	144.21	144.21	117.27	0.000
X3 × X3	1	13.32	13.32	10.83	0.022
Interactions	3	794.60	264.87	215.37	0.000
X1 × X2	1	73.33	73.33	59.63	0.001
X1 × X3	1	703.92	703.92	572.39	0.000
X2 × X3	1	17.34	17.34	14.10	0.013
Residual error	5	6.15	1.23		
Lack of fit	3	5.90	1.97	16.04	0.059
Pure error	2	0.25	0.12		
Total	14	5693.88			

**Table 4:** Analysis of Variance (ANOVA) of total flavonoid content.

Source	DF	Adjusted SS	Adjusted SM	F-Value	*p*-Value
Model	9	7.77565	0.86396	7.85	0.018
Linear	3	1.87395	0.62465	5.67	0.046
X_1_	1	1.48092	1.48092	13.45	0.014
X_2_	1	0.12475	0.12475	1.13	0.336
X_3_	1	0.26828	0.26828	2.44	0.179
Quadratic	3	3.83471	1.27824	11.61	0.011
X_1_ × X_1_	1	3.80516	3.80516	34.57	0.002
X_2_ × X_2_	1	0.01134	0.01134	0.10	0.761
X_3_ × X_3_	1	0.00034	0.00034	0.00	0.958
Interactions	3	2.06699	0.68900	6.26	0.038
X_1_ × X_2_	1	0.29214	0.29214	2.65	0.164
X_1_ × X_3_	1	1.61163	1.61163	14.64	0.012
X_2_ × X_3_	1	0.16322	0.16322	1.48	0.278
Residual error	5	0.55041	0.11008		
Lack of fit	3	0.35714	0.11905	1.23	0.477
**Pure error**	2	0.19327	0.09664		
**Total**	14	8.32606			

**Table 5:** Analysis of Variance (ANOVA) of ABTS scavenging activity.

Source	DF	Adjusted SS	Adjusted SM	F-Value	*p*-Value
Model	9	0.324838	0.036093	22.32	0.002
Linear	3	0.026771	0.008924	5.52	0.048
X1	1	0.013203	0.013203	8.17	0.036
X2	1	0.000120	0.000120	0.07	0.796
X3	1	0.013448	0.013448	8.32	0.034
Quadratic	3	0.243813	0.081271	50.26	0.000
X1 × X1	1	0.174335	0.174335	107.81	0.000
X2 × X2	1	0.023754	0.023754	14.69	0.012
X3 × X3	1	0.027600	0.027600	17.07	0.009
Interactions	3	0.054254	0.018085	11.18	0.012
X1 × X2	1	0.019740	0.019740	12.21	0.017
X1 × X3	1	0.034225	0.034225	21.17	0.006
X2 × X3	1	0.000289	0.000289	0.18	0.690
Residual error	5	0.008085	0.001617		
Lack of fit	3	0.007644	0.002548	11.56	0.081
Pure error	2	0.000441	0.000220		
Total	14	0.332923			

**Table 6:** Analysis of Variance (ANOVA) of anti-S*. aureus* activity.

Source	DF	Adjusted SS	Adjusted SM	F-Value	*p*-Value
Model	9	449.219	49.913	12.78	0.006
Linear	3	175.781	58.594	15.00	0.006
X1	1	78.125	78.125	20.00	0.007
X2	1	78.125	78.125	20.00	0.007
X3	1	19.531	19.531	5.00	0.076
Quadratic	3	78.125	26.042	6.67	0.034
X1 × X1	1	36.058	36.058	9.23	0.029
X2 × X2	1	0.000	0.000	0.00	1.000
X3 × X3	1	36.058	36.058	9.23	0.029
Interactions	3	195.312	65.104	16.67	0.005
X1 × X2	1	39.063	39.063	10.00	0.025
X1 × X3	1	156.250	156.250	40.00	0.001
X2 × X3	1	0.000	0.000	0.00	1.000
Residual error	5	19.531	3.906		
Lack of fit	3	19.531	6.510		
Pure error	2	0.000	0.000		
Total	14	468.750			

**Table 7:** Analysis of Variance (ANOVA) of anti-E*. coli* activity.

Source	DF	Adjusted SS	Adjusted SM	F-Value	*p*-Value
Model	9	570.313	63.368	8.11	0.016
Linear	3	117.188	39.063	5.00	0.058
X1	1	78.125	78.125	10.00	0.025
X2	1	19.531	19.531	2.50	0.175
X3	1	19.531	19.531	2.50	0.175
Quadratic	3	257.812	85.937	11.00	0.012
X1 × X1	1	81.130	81.130	10.38	0.023
X2 × X2	1	81.130	81.130	10.38	0.023
X3 × X3	1	81.130	81.130	10.38	0.023
Interactions	3	195.313	65.104	8.33	0.022
X1 × X2	1	39.062	39.062	5.00	0.076
X1 × X3	1	156.250	156.250	20.00	0.007
X2 × X3	1	0.000	0.000	0.00	1.000
Residual error	5	39.062	7.812		
Lack of fit	3	39.062	13.021	*	*
Pure error	2	0.000	0.000		
Total	14	609.375			

*: Not applicable, the pure error was zero therefore the lack-of-fit could not be computed.

**Table 8:** Validation of the optimized extraction conditions by comparing predicted and experimental values of total phenolic content (TPC), total flavonoid content (TFC), antioxidant activity (ABTS IC_50_), and antibacterial activities of *Opuntia stricta* extract.

Response	Predicted Optimal Value	Experimental Optimal Value	Relative Error (%)
TPC (mg GAE/g)	77.194	77.23 ± 0.055	0.05
TFC (mg CE/g)	3.787	3.741 ± 0.060	1.23
ABTS (IC_50_ mg/mL)	0.187	0.190 ± 0.007	1.58
Anti-*S. aureus* (mg/mL)	5.476	5.00	9.52
Anti-*E. coli* (mg/mL)	7.883	10.00	21.17

±: Standard deviation of three replicates.

**Table 9:** Chemical composition of the optimized whole-fruit extract of *Opuntia stricta* determined by HPLC analysis.

N°	Compounds	Concentration (mg/g of Extract)	Retention Time (min)
1	Non-identified	-	6.000
2	Non-identified	-	7.794
3	3,4-Dihydroxyphenylacetic acid (DOHPAC)	0.14	9.555
4	Non-identified	-	9.997
5	Catechin	0.43	10.990
6	4-Hydroxyphenylacetic acid (HPAC)	14.86	11.842
7	Epicatechin	6.05	12.456
8	4-Hydroxy-3-methoxybenzoic acid (Vanillic acid)	0.43	12.657
9	Non-identified	-	12.671
10	Non-identified	-	15.813

**Table 10:** Free energies of binding (ΔG) of the docked compounds against the selected targets, expressed in Kcal/mol.

Compound/Target	PPK of *E. coli*	FnBPA of *S. aureus*
3,4-Dihydroxyphenylacetic acid	−6.0	−6.4
Catechin	−8.2	−7.9
4-Hydroxyphenylacetic acid	−6.2	−5.7
Epicatechin	−8.1	−7.9
4-Hydroxy-3-methoxybenzoic acid (Vanillic acid)	−5.5	−5.8

## Data Availability

The datasets generated during the current study are available from the corresponding author upon reasonable request.

## Supplementary Materials

The following supporting information can be downloaded at: https://www.mdpi.com/article/10.3390/foods15193557/s1, Figure S1. HPLC-DAD chromatogram illustrating the profile of Chlorogenic acid detected at 330 nm; Figure S2. HPLC-DAD chromatogram illustrating the profile of Vanillic acid detected at 254 nm; Figure S3. HPLC-DAD chromatogram illustrating the profile of Caffeic acid detected at 330 nm; Figure S4. HPLC-DAD chromatogram illustrating the profile of Apigenin detected at 330 nm; Figure S5. HPLC-DAD chromatogram illustrating the profile of Proto-catechic acid detected at 254 nm; Figure S6. HPLC-DAD chromatogram illustrating the profile of Tyrosol detected at 280 nm; Figure S7. HPLC-DAD chromatogram illustrating the profile of Catechin detected at 280 nm; Figure S8. HPLC-DAD chromatogram illustrating the profile of Trans-cinnamic acid detected at 280 nm; Figure S9. HPLC-DAD chromatogram illustrating the profile of Epicatechin detected at 280 nm; Figure S10. HPLC-DAD chromatogram illustrating the profile of rutin detected at 254 nm; Figure S11. HPLC-DAD chromatogram illustrating the profile of Esculin detected at 330 nm; Figure S12. HPLC-DAD chromatogram illustrating the profile of Esculitin detected at 330 nm; Figure S13. HPLC-DAD chromatogram illustrating the profile of DOHPAC detected at 280 nm; Figure S14. HPLC-DAD chromatogram illustrating the profile of Catechol detected at 280 nm; Figure S15. HPLC-DAD chromatogram illustrating the profile of HPAC detected at 280 nm.
