# Supplementary material for: Integrated Optimization of Opuntia stricta Extracts Coupled with HPLC-Based Phenolic Profiling: Linking Phytochemical Composition to Molecular Docking Simulation
Source: Foods. 2026 Oct 7;15(19):3557. doi: 10.3390/foods15193557 (PMC13665341; doi:10.3390/foods15193557)
Supplement: Supplementary file 1 [file foods-15-03557-s001.zip › foods-4555825-supplementary.pdf]

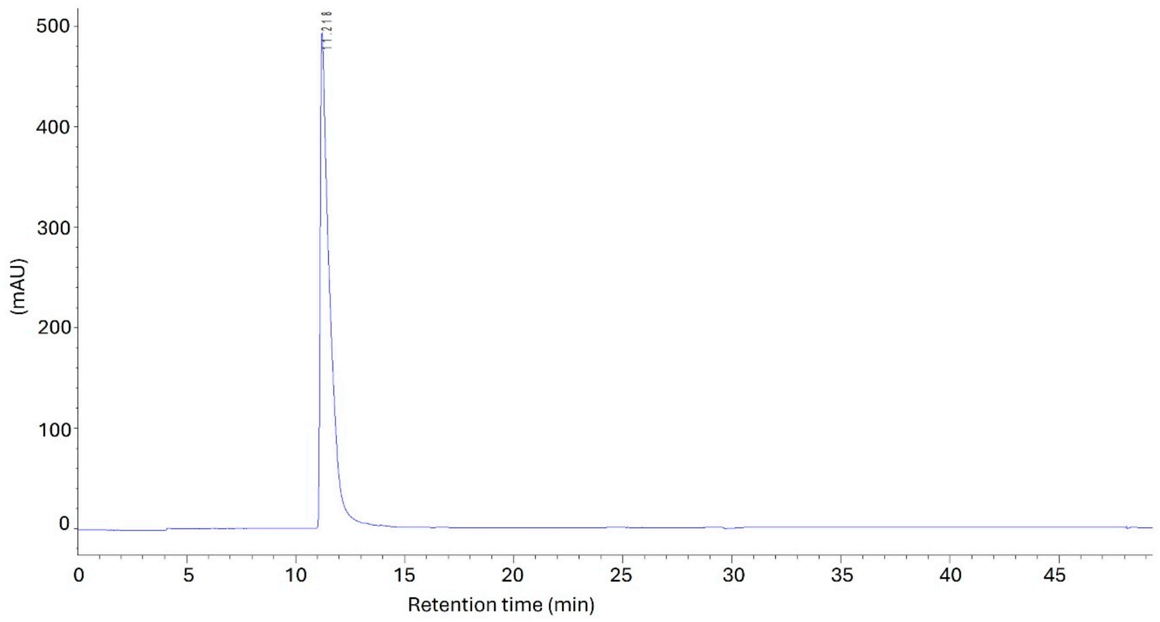

Figure S1. HPLC-DAD chromatogram illustrating the profile of *Chlorogenic acid* detected at 330 nm.

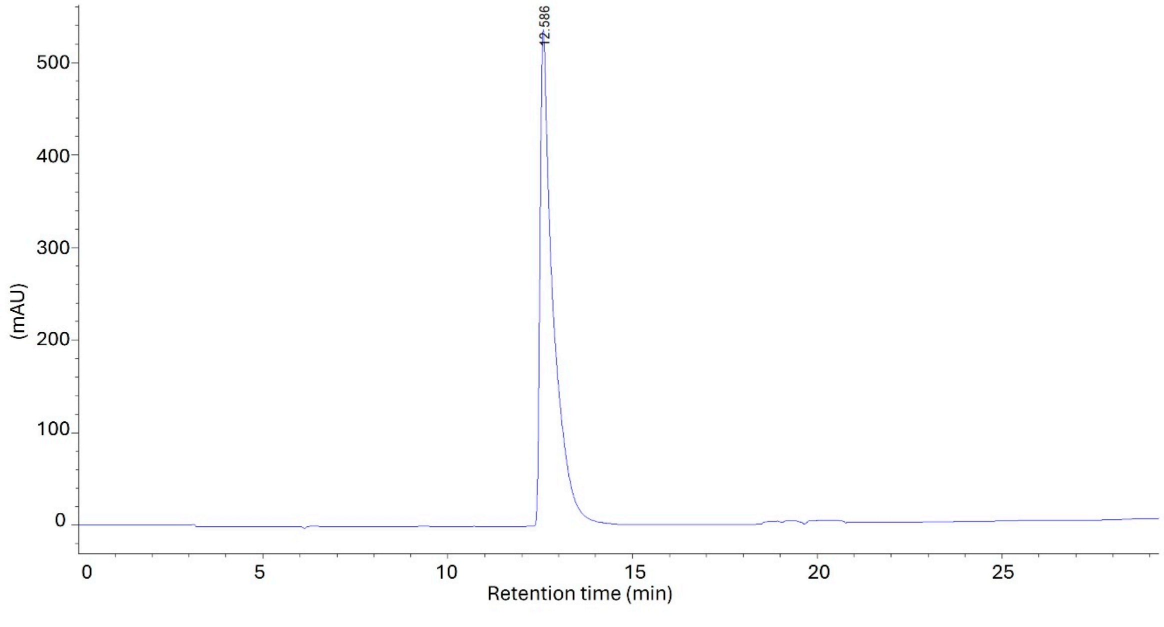

Figure S2. HPLC-DAD chromatogram illustrating the profile of *Vanillic acid* detected at 254 nm.

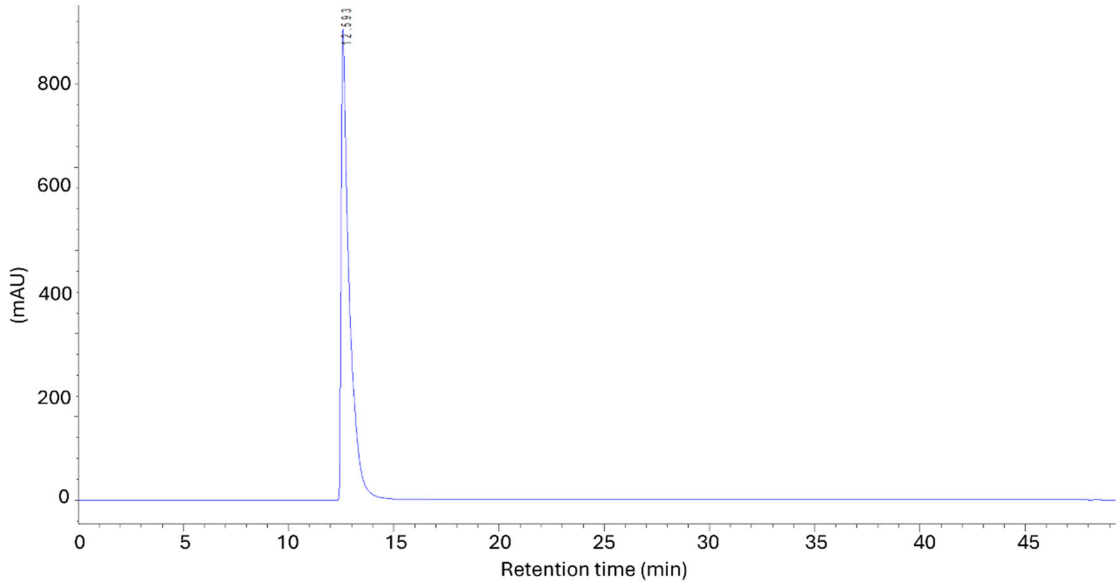

Figure S3. HPLC-DAD chromatogram illustrating the profile of *Caffeic acid* detected at 330nm.

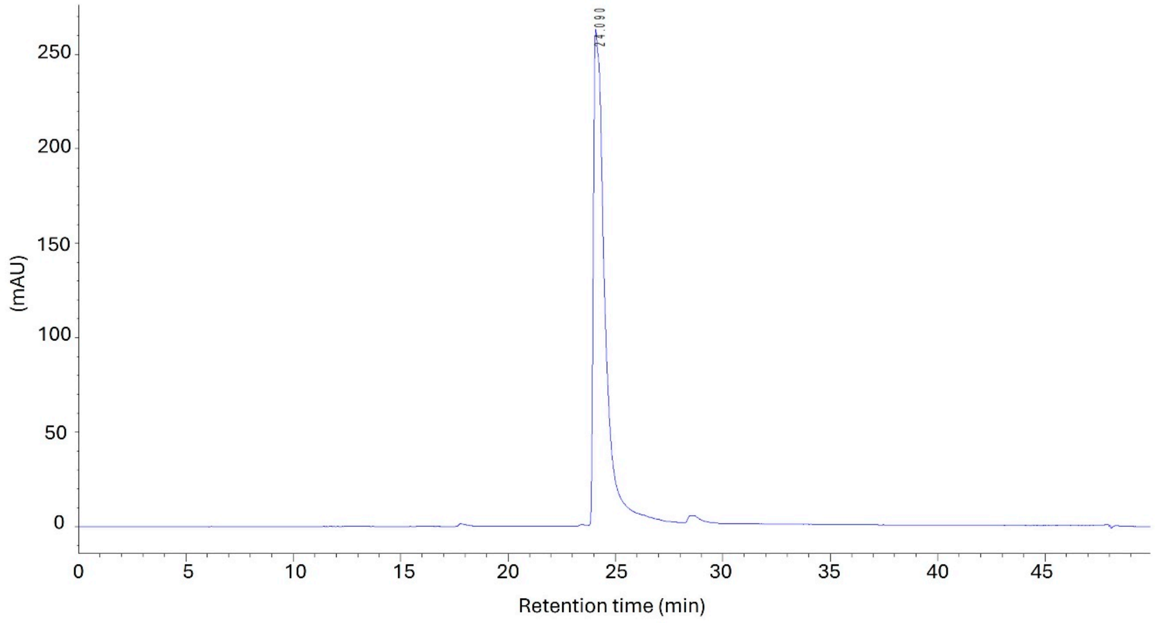

Figure S4. HPLC-DAD chromatogram illustrating the profile of Apigenin detected at 330nm.

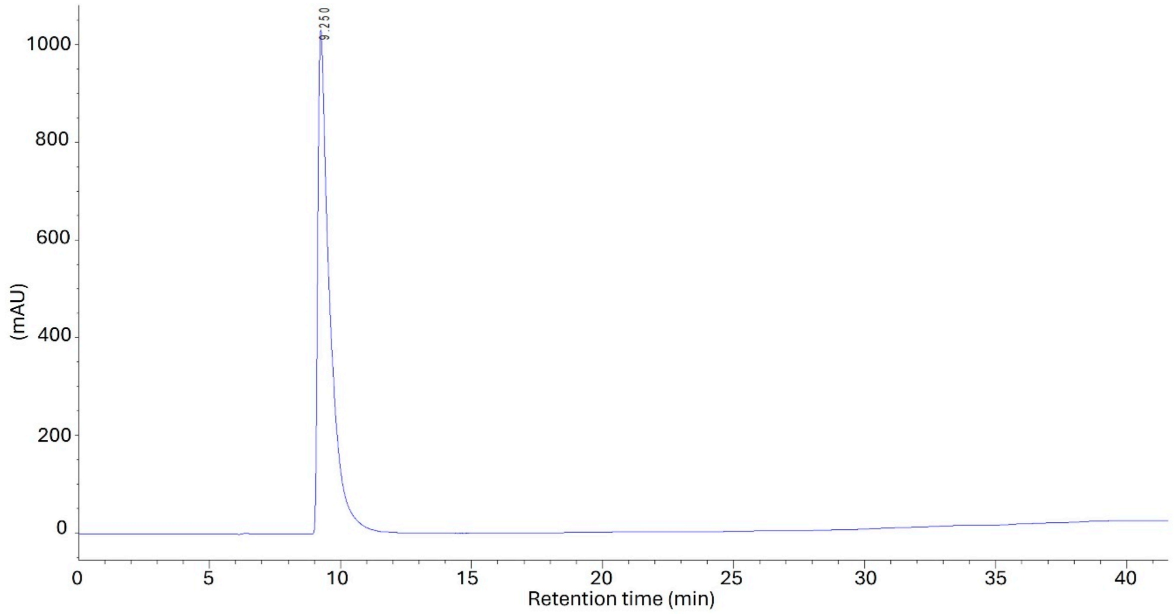

Figure S5. HPLC-DAD chromatogram illustrating the profile of Proto-catechic acid detected at 254nm.

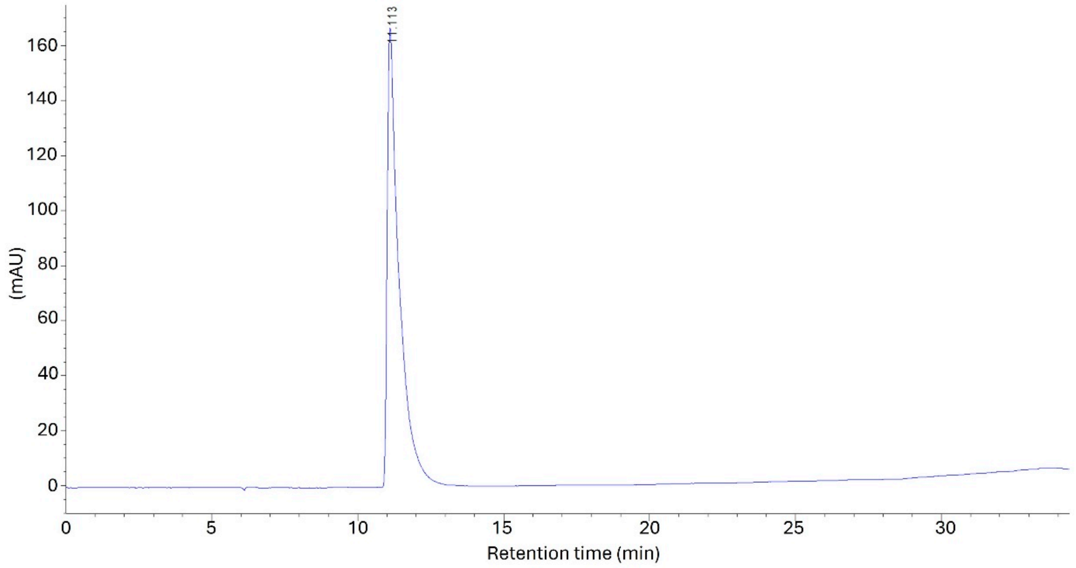

Figure S6. HPLC-DAD chromatogram illustrating the profile of Tyrosol detected at 280 nm.

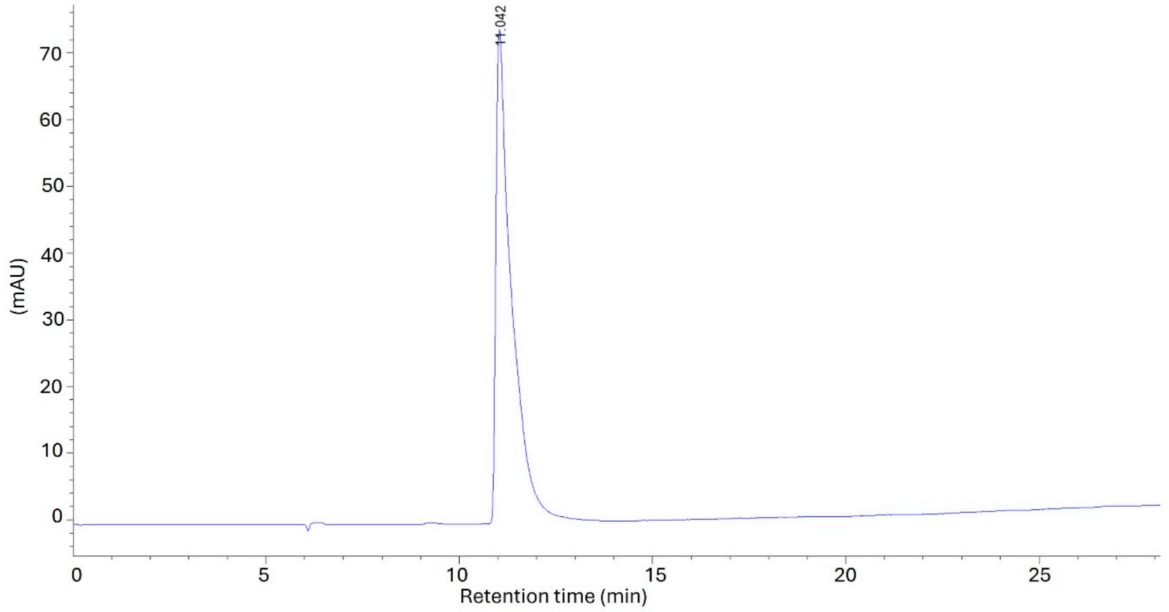

Figure S7. HPLC-DAD chromatogram illustrating the profile of Catechin detected at 280nm.

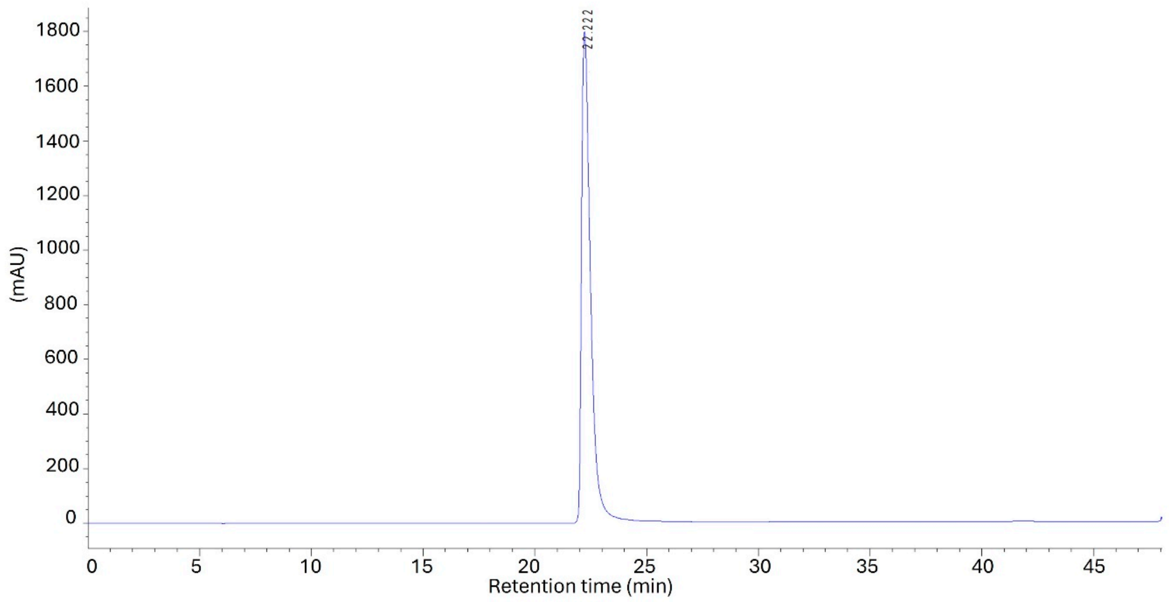

Figure S8. HPLC-DAD chromatogram illustrating the profile of Trans-cinnamic acid detected at 280 nm.

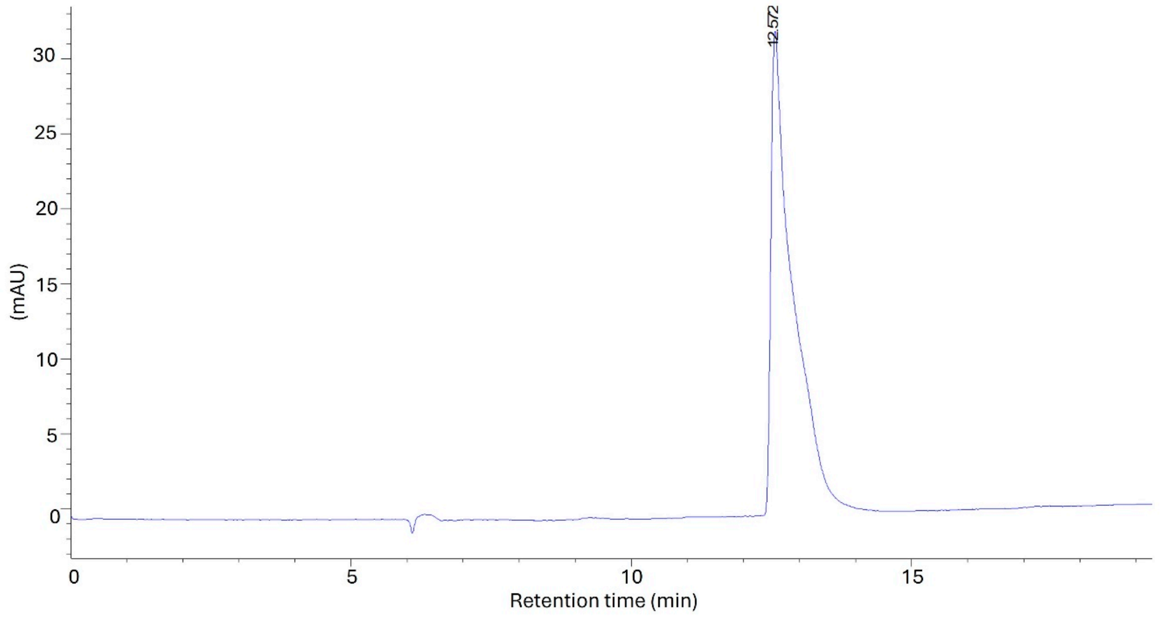

Figure S9. HPLC-DAD chromatogram illustrating the profile of Epicatechin detected at 280nm.

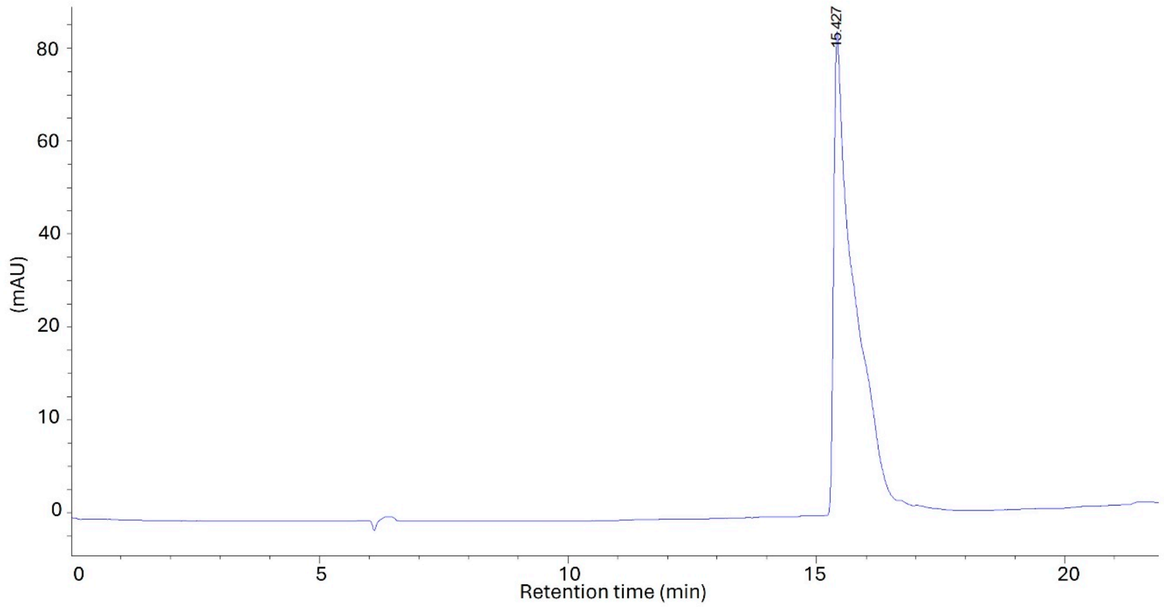

Figure S10. HPLC-DAD chromatogram illustrating the profile of rutin detected at 254 nm.

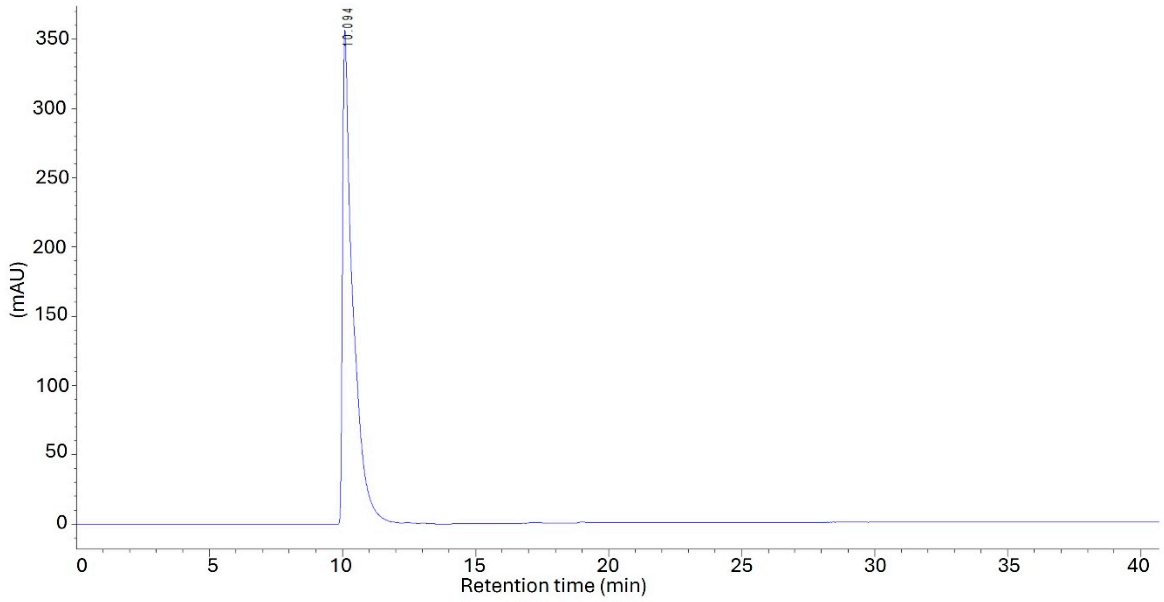

Figure S11. HPLC-DAD chromatogram illustrating the profile of Esculin detected at 330nm.

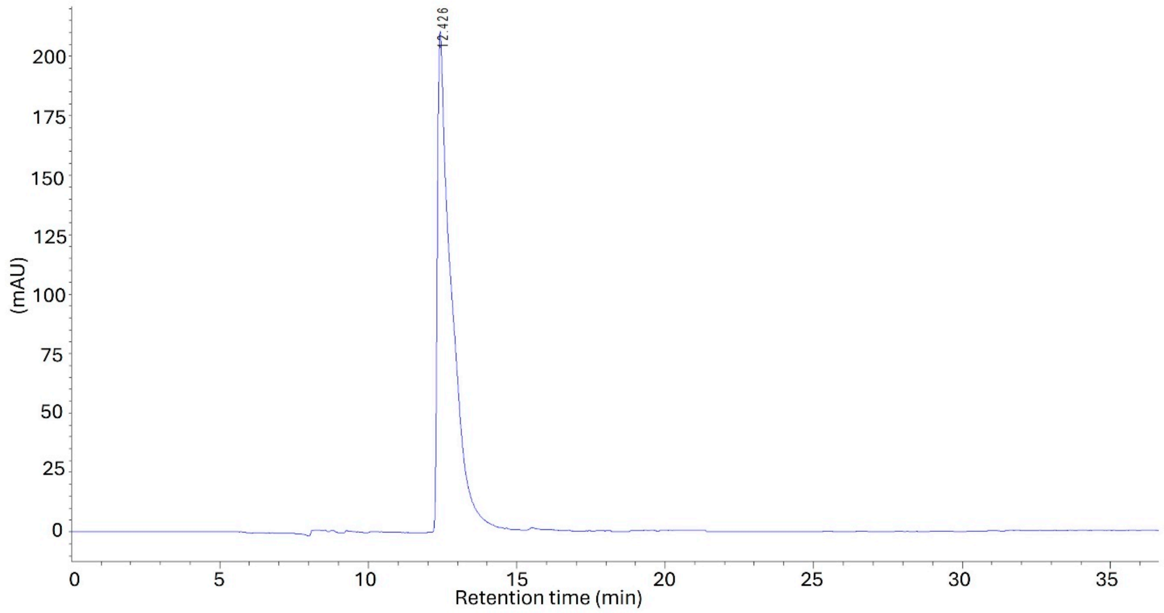

Figure S12. HPLC-DAD chromatogram illustrating the profile of Esculitin detected at 330nm.

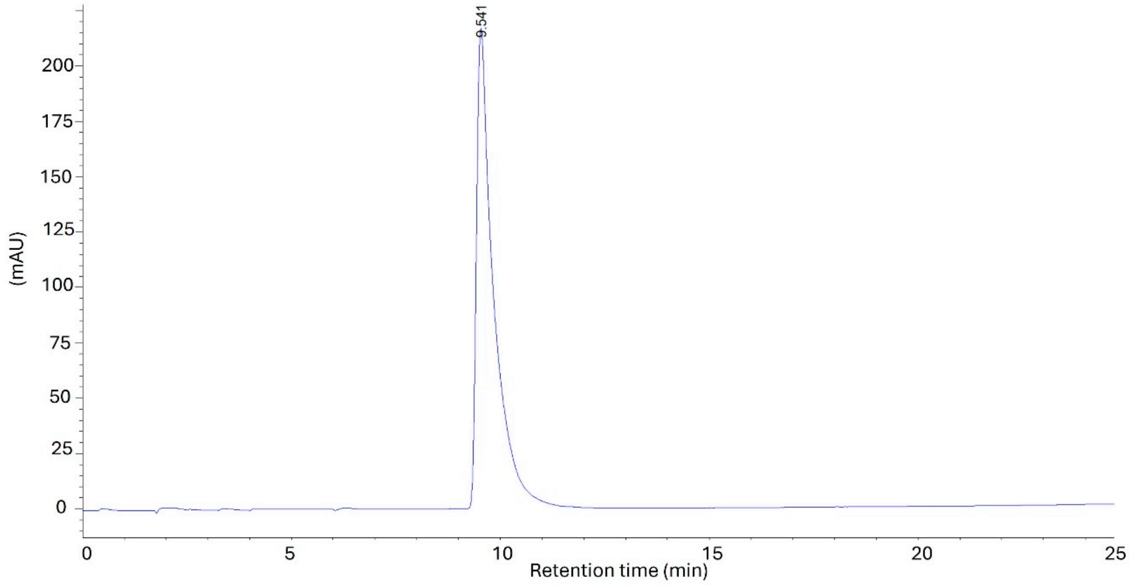

Figure S13. HPLC-DAD chromatogram illustrating the profile of DOHPAC detected at 280nm.

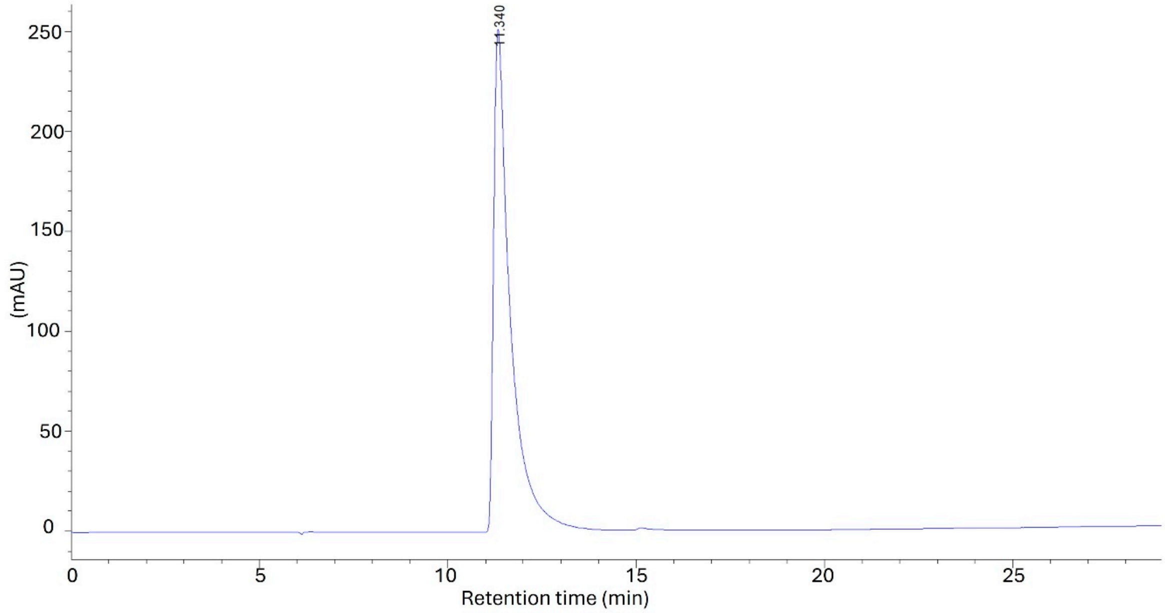

Figure s14. HPLC-DAD chromatogram illustrating the profile of Catechol detected at 280nm.

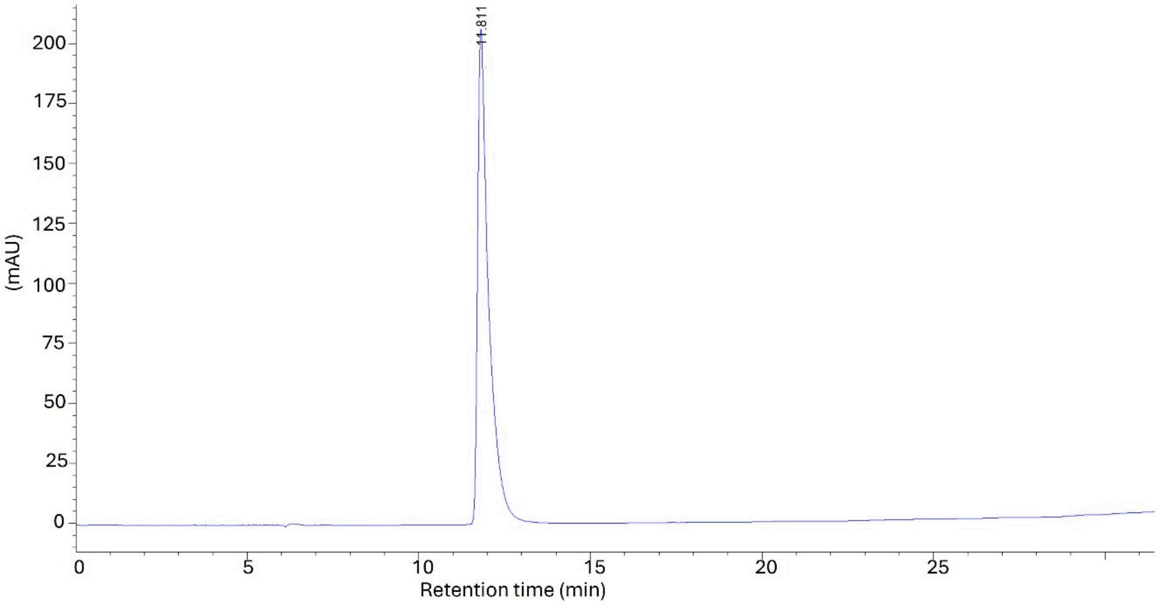

Figure S15. HPLC-DAD chromatogram illustrating the profile of HPAC detected at 280nm.
